# Metabolomic analysis for disclosing nutritional and therapeutic prospective of traditional rice cultivars of Cauvery deltaic region, India

**DOI:** 10.3389/fnut.2023.1254624

**Published:** 2023-09-28

**Authors:** Dhandayuthapani Udhaya Nandhini, Subramanian Venkatesan, Kandasamy Senthilraja, Ponnusamy Janaki, Balasubramaniam Prabha, Sadasivam Sangamithra, Sivaprakasam Jidhu Vaishnavi, Sadasivam Meena, Natarajan Balakrishnan, Muthurajan Raveendran, Vellingiri Geethalakshmi, Eagan Somasundaram

**Affiliations:** ^1^Centre of Excellence in Sustaining Soil Health, Anbil Dharmalingam Agricultural College and Research Institute, Trichy, Tamil Nadu, India; ^2^Directorate of Research, Tamil Nadu Agricultural University, Coimbatore, Tamil Nadu, India; ^3^Directorate of Crop Management, Tamil Nadu Agricultural University, Coimbatore, Tamil Nadu, India; ^4^Nammazhvar Organic Farming Research Centre, Tamil Nadu Agricultural University, Coimbatore, Tamil Nadu, India; ^5^Department of Renewable Energy Engineering, Tamil Nadu Agricultural University, Coimbatore, Tamil Nadu, India; ^6^Department of Agricultural Entomology, Tamil Nadu Agricultural University, Coimbatore, Tamil Nadu, India; ^7^Amrita School of Agricultural Sciences, Coimbatore, Tamil Nadu, India; ^8^Agro-Climatic Research Centre, Tamil Nadu Agricultural University, Coimbatore, Tamil Nadu, India; ^9^Agribusiness Development, Tamil Nadu Agricultural University, Coimbatore, Tamil Nadu, India

**Keywords:** grain metabolome, traditional rice varieties, gas chromatography-mass spectrometry, univariate and multivariate analysis, hierarchical clustering

## Abstract

Traditional rice is gaining popularity worldwide due to its high nutritional and pharmaceutical value, as well as its high resistance to abiotic and biotic stresses. This has attracted significant attention from breeders, nutritionists, and plant protection scientists in recent years. Hence, it is critical to investigate the grain metabolome to reveal germination and nutritional importance. This research aimed to explore non-targeted metabolites of five traditional rice varieties, *viz*., Chinnar, Chithiraikar, Karunguruvai, Kichili samba, and Thooyamalli, for their nutritional and therapeutic properties. Approximately 149 metabolites were identified using the National Institute of Standards and Technology (NIST) library and Human Metabolome Database (HMDB) and were grouped into 34 chemical classes. Major classes include fatty acids (31.1–56.3%), steroids and their derivatives (1.80–22.4%), dihydrofurans (8.98–11.6%), prenol lipids (0.66–4.44%), organooxygen compounds (0.12–6.45%), benzene and substituted derivatives (0.53–3.73%), glycerolipids (0.36–2.28%), and hydroxy acids and derivatives (0.03–2.70%). Significant variations in metabolite composition among the rice varieties were also observed through the combination of univariate and multivariate statistical analyses. Principal component analysis (PCA) reduced the dimensionality of 149 metabolites into five principle components (PCs), which explained 96% of the total variance. Two clusters were revealed by hierarchical cluster analysis, indicating the distinctiveness of the traditional varieties. Additionally, a partial least squares-discriminant analysis (PLS-DA) found 17 variables important in the projection (VIP) scores of metabolites. The findings of this study reveal the biochemical intricate and distinctive metabolomes of the traditional therapeutic rice varieties. This will serve as the foundation for future research on developing new rice varieties with traditional rice grain metabolisms to increase grain quality and production with various nutritional and therapeutic benefits.

## 1. Introduction

Rice is the most important staple crop after wheat, and two-thirds of the world's population relies on rice as their main food supply (1). Globally, rice is cultivated on 167.2 million acres, and the current rice production is 769.4 million tons (2). In India, rice is cultivated on 45 million hectares of land with a production of 125 million tons (3), and West Bengal, Uttar Pradesh, Punjab, Odisha, Andhra Pradesh, Telangana, Chhattisgarh, Bihar, Tamil Nadu, Assam, and Madhya Pradesh are the major rice growing states in India. In India, rural communities typically eat traditional rice varieties with coarse grains, which have lower production costs than newer rice varieties with high-quality grains. After COVID-19, the eating habits of humans have been changed much, and all want to have quality and immune-boosting food grains. One of the food grains gaining attention in our diet is traditional rice varieties as they are rich in nutrients and bioactive compounds such as polyphenols, phytochemicals, antioxidants, vitamins, and minerals. Hence, the demand for traditional rice varieties in India has increased due to their higher nutritional qualities, which also help protect us from lifestyle disorders (4).

Tamil Nadu, a southern state of India, is home to ~400 traditional rice varieties, and very specifically, the Cauvery deltaic region is known for the collection of traditional rice germplasm. The varieties such as Kichili samba, Poongar, Thooyamalli, Chinnar, Karunguruvai, Milagu samba, Seeraga samba, Kaiviral samba, Mappillai samba, Karuppu kavuni, Kattuyanam, and Kuzhiyadichan are popular among them. These varieties have been especially preferred for their diuretic and anti-inphemmatory effects (5). Along with being an infant's first solid food, rice is also crucial to the baby shower celebration (6). For instance, in Tamil Nadu, a specific variety of rice called Mappillai samba is provided to the groom to increase fertility (7). The antioxidant potential of some medicinal rice varieties such as Kattuyanam, Mappillai samba, Navara, Karunguruvai, Kavuni, Kichadi samba, Illupai poo samba, Kalanamak, Garudan samba, and Seeraga samba can treat human ailments and cause various physiological changes in the human body (4). Karunguruvai is used for healing elephantiasis joint pain, chickenpox, Hansen's illness, cholera, skin sickness, venomous bites, urinary infections, and canine nibbles, and weakens bad cholesterol (8). However, the pharmaceutical values of these varieties may vary based on their individual genetic makeup.

The bioactive compounds present in rice include primary metabolites (sugars, lipids) and secondary metabolites (terpenoids, steroids, hydrocarbons, etc.) which are known to possess pharmaceutical and health-promoting values in addition to anticancer, anti-atherogenic, and blood sugar and cholesterol-regulating activities (9). However, the composition, characteristics, and functions of these phytochemicals in traditional rice varieties have not yet been completely explored and documented. This highlights the need for further study to describe these bioactive substances and enable their potential use.

Untargeted metabolomics provides comprehensive metabolite coverage, and the generated data can be utilized to generate or test hypotheses. It is not necessary to have prior knowledge of treatment-responsive compounds, and the findings can be utilized as a screening tool to direct further focused investigation of compounds and/or interest pathways (10). For instance, Rajagopalan et al. (11) found phytosterols in Mappillai samba that possess cholesterol-lowering, antioxidant, and anticancer properties. Ashokkumar et al. (12, 13) reported linoleic and oleic acids in Kaiviral samba. Sukhonthrea et al. (14) identified myristic acid, nonanal, (*E*)-β-ocimene, and 6,10,14-trimethyl-2-pentadenone in red rice and myristic acid, nonanal, caproic acid, pentadecanal, and pelargonic acid in black pigmented rice. Kotamreddy et al. (15) identified l-threonine, l-aspartic acid, tyrosol, hydroxytyrosol, 4-hydroxybenzoic acid, 4-coumaric acid, isoferulic acid, azelaic acid, and galactitol in rice. The bioactive features in rice bran include nutritional components such as hemicellulose, cellulose, arabinoxylan, polyphenolics, pectin, lignin, β-glucan, β-sitosterol, γ-oryzanol, vitamin E isomers, vitamin B9, essential amino acids, and micronutrients (16, 17). The beneficial secondary metabolites found in rice bran include dietary fiber, phytosteroids, oryzanol, tocopherol, tocotrienols, ferulic acids, and other phenolic compounds. Hence, in this study, we used brown rice to retain all bioactive compounds. According to reports, rice bran lowers blood and liver cholesterol, inhibits platelet aggregation, increases skin microcirculation, and prevents the development of ulcers (18).

In the current study, a triple quadrupole mass spectrometry-based gas chromatography (GC-MS/MS) metabolomics technique was used to examine the kinds and relative amounts of non-targeted-based primary and secondary metabolites in five traditional varieties, *viz*., Chinnar, Chithiraikar, Karunguruvai, Kichili samba, and Thooyamalli. Chemometric analyses such as principle component and partial least square discrimination analysis were used to classify the sample as diverse. Hierarchical cluster analysis and metabolic pathway identification were carried out to categorize the functional metabolites that possess a health advantage. The findings will offer a theoretical framework for the development of functional foods using traditional rice and identify molecular markers for deciding breeding programs.

## 2. Materials and methods

The traditional rice varieties, *viz*., Chinnar, Chithiraikar, Karunguruvai, Kichili samba, and Thooyamalli, were selected for the study, and a legal permission letter for the collection of plant material adhering to institutional, national, and international guidelines and legislation was obtained from the Director of Research of the Tamil Nadu Agricultural University, Coimbatore, Tamil Nadu, India. The varieties were obtained from the local farmers of the Cauvery delta region of Tamil Nadu, India. They were grown with canal irrigation during the *Samba* season (July–December 2021) by adopting the required agronomic practices and harvested at physiological maturity. The growth location, agronomic practices, and harvest time were similar in the entire sampled farmer's field. These was given high significance as they will directly alter the chemical composition of grains. Detailed agronomical characteristics of the selected rice varieties are given in Table 1. Three biological replicates of samples were collected manually. Approximately 500 g of each variety was cleaned by removing foreign matter such as stones, straw, and dirt from each replication and sun-dried until the moisture content reached 12.0%. All the lab experiments were carried out in the Laboratory of the Center of Excellence in Soil Health, Anbil Dharmalingam Agricultural College and Research Institute, Tamil Nadu Agricultural University Campus, Trichy, Tamil Nadu, India, in January 2022.

**Table 1:** Detailed agronomic characteristics of the selected traditional rice.

**Name of the variety**	**Karunguruvai**	**Thooyamalli**	**Chinnar**	**Chithiraikar**	**Kichili samba**
Origin	Tamil Nadu, India	Tamil Nadu, India	Tamil Nadu India	Tamil Nadu, India	Tamil Nadu, India
Pedigree	Unknown	Unknown	Unknown	Unknown	Unknown
Duration (Days)	120–125	135–140	110–120	110–115	140–145
Average height (cm)	95	115	85	165	105
Number of grains per ear head	85–90	140–145	135–140	210–215	130–135
Yield of grain (kg acre^−1^)	825	1,125	2,700	1,000	1,125
Yield of straw (kg acre^−1^)	1,200	1,050	2,550	950	1,050
1,000 grain weight (g)	25	27	21	33	17
Color of pericarp	Red	White	White	Red	White

### 2.1. Sample preparation

One gram of the finely ground rice sample was placed in a 20 ml centrifuge tube and 10 ml of HPLC-grade ethanol was added. The tube was then vortexed (LABOID International, Himachal Pradesh, India) at 2,000 rpm for 10 min. The mixture was centrifuged at 5,000 rpm for 20 min (19). The supernatant was concentrated in a rotary evaporator and filtered using a 0.2 μm PVDF syringe filter. The filtrate was kept in an airtight glass vial at 4°C for chromatographic analysis.

### 2.2. Chromatography condition and analysis

The filtered ethanolic extract was subjected to metabolite analysis in a Thermo Fisher ISQ triple quadrupole gas chromatograph-mass spectrometer (Thermo Fisher TSQ 8000 Duo Triple Quadrupole GC-MS/MS). The GC was equipped with a fused silica capillary column DB-5 ms (30 m, 0.25 mm ID) with a film thickness of 0.25 μm. Helium was used as a carrier gas at a flow rate of 1.0 ml/min. Then, 1 ml of the sample was kept in a 2-ml screw-top vial in an auto-injector, and 1 μl of the sample was injected in split mode (1:10). The detector and injector temperature were maintained at 250°C. The oven temperature was programmed as 70°C for 15 min to 280°C at 30°C/min (10 min hold), up to 250°C at 10°C per min. The MS conditions were full scan mode, electron impact spectra at 70 eV, ion source temperature of 260°C, and transmission line temperature of 280°C. The mass scan range (*m*/*z*) was 50–650 amu with a solvent delay of 3 min (12). The bioactive molecules were identified by comparing mass spectra with the NIST 08 Mass Spectra Library (National Institute of Standards and Technology). The name, molecular weight, and structure were ascertained from the NIST, PubChem, and HMDB databases.

### 2.3. Statistical analysis

All the experiments were performed in triplicate. The online MetaboAnalyst 5.0 (20) software was used for the statistical analysis of the data. For univariate and multivariate analyses, the mean data were used to normalize the relative amounts of data for identified metabolites. A one-way ANOVA was performed on the dataset, followed by *post-hoc* Tukey's honestly significant difference (HSD) test with an adjusted *p*-value of 0.05 and a Student's *t*-test (*P* ≤ 0.05). The unsupervised approach of pattern identification known as PCA was first used to investigate the data matrix's inherent variation. Following that, the dataset's discriminating molecular characteristics were removed using PLS-DA, a supervised classification method.

These data were ordered according to their abundance value, and the most distinctive features were chosen for additional data clustering by extracting the most information from potentially discriminating features. In order to analyze and illustrate the similarities between sample replicates and to better understand the differences in metabolite compounds among different sample types, a hierarchical clustering analysis (HCA) dendrogram with heatmap representation was built. The dissimilarity was measured using the Euclidean distance, and the clusters were described using Ward's approach.

## 3. Results

**Figure 1:**
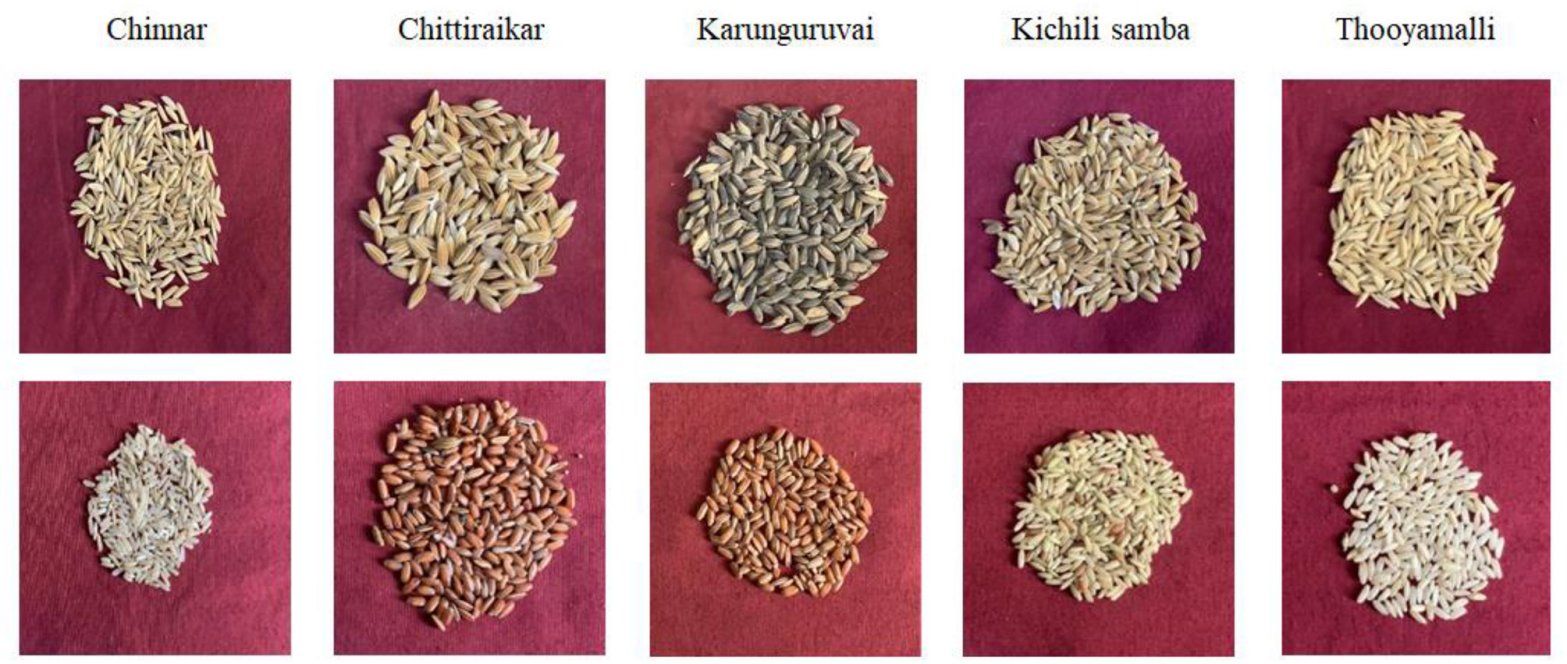
Photograph showing the selected traditional rice grains.

The five traditional rice varieties currently grown in Tamil Nadu, *viz*., Chinnar, Chithiraikar, Karunguruvai, Kichili samba, and Thooyamalli, were subjected to metabolome analysis using GC-MS/MS. Chithiraikar and Karunguruvai are pigmented types, while Chinnar, Kichili samba, and Thooyamalli are white-colored rice varieties (Figure 1). Non-targeted metabolomic analysis was employed to detect the metabolites in these rice varieties to fully understand the variations in their chemical composition. Chromatographic analysis of the grain metabolome detected about 149 compounds (Supplementary Figures S1–S5 and Supplementary Table S1) from the five traditional rice varieties, which were identified and categorized into 34 chemical classes using the NIST library and HMDB database. The identified 149 metabolites comprise 7 benzene and its substituted derivatives, 9 saturated hydrocarbons, 14 prenol lipids, 20 organooxygen compounds, 25 steroids and steroid derivatives, and 39 fatty acyls. The classes such as hydroxy acids and derivatives, phenols, purine nucleosides, quinoline derivatives, and unsaturated hydrocarbons hold two metabolites under each class (Table 2), and the remaining 22 classes share one metabolite under each category.

**Table 2:** Major classes of metabolites identified in the five traditional rice varieties and its concentration (area %).

**Class**	**Chinnar**	**Chithiraikar**	**Karunguruvai**	**Kichili samba**	**Thooyamalli**
Acyl halides (1)	-	-	1.08	-	-
Alkaloids and derivatives (1)	-	-	-	0.05	-
Alkyl halides (1)	2.89	-	-	-	-
Aspidosperma tan-type alkaloids (1)	0.6	0.06	0.06	0.01	0.02
Azoles (1)	-	-	0.07	-	0.07
Benzene and substituted derivatives (7)	1.95	0.53	3.73	-	-
Benzothiazoles (1)	3.17	-	1.23	-	-
Carbothioic S-acids (1)	0.02	-	-	-	-
Carboxylic acids and derivatives (3)	0.01	-	0.59	-	-
Coumarins and derivatives (1)	-	0.02	1.17	0.06	-
Dihydrofurans (1)	8.98	-	10.23	11.62	15.5
Dioxanes (1)	0.02	-	-	0.01	-
Dioxolanes (1)	0.25	-	0.01	-	0.42
Fatty Acyls (21)	31.14	56.32	39.08	47.73	50.54
Furans (1)	-	-	0.26	-	-
Furofurans (1)	-	-	0.16	-	-
Glycerolipids (1)	1.78	1.75	-	0.36	2.28
Hydroxy acids and derivatives (1)	1.51	0.9	0.03	-	2.7
Imidazopyrimidines (1)	0.02	-	0.02	-	0.08
Lupin alkaloids (1)	-	0.26	-	-	-
Naphthopyrans (1)	0.71	-	-	-	-
Organic phosphonic acids and derivatives (1)	-	-	-	0.07	-
Organonitrogen compounds (1)	-	-	0.12	-	-
Organooxygen compounds (22)	0.32	0.32	6.45	0.66	1.33
Phenols (2)	0.23	0.18	0.37	0.12	0.35
Prenol lipids (15)	4.44	2.06	0.66	1.48	1.24
Purine nucleosides (2)	-	-	2.35	-	-
Pyridines and derivatives (1)	-	-	-	0.06	-
Quinolines and derivatives (2)	-	-	0.1	0.05	-
Saturated hydrocarbons (9)	1.05	0.63	0.11	-	0.59
Steroids and steroid derivatives (23)	5.72	22.93	-	20.35	9.69
Thiols (1)	-	-	0.3	-	-
Triazole ribonucleosides and ribonucleotides (1)	-	0.15	-	-	-
Unsaturated hydrocarbons (2)	-	-	-	0.38	1.03
Total compounds yield (%)	64.81	86.11	67.1	82.96	85.84

^*^Number of compounds in each class is mentioned in parenthesis.

**Figure 2:**
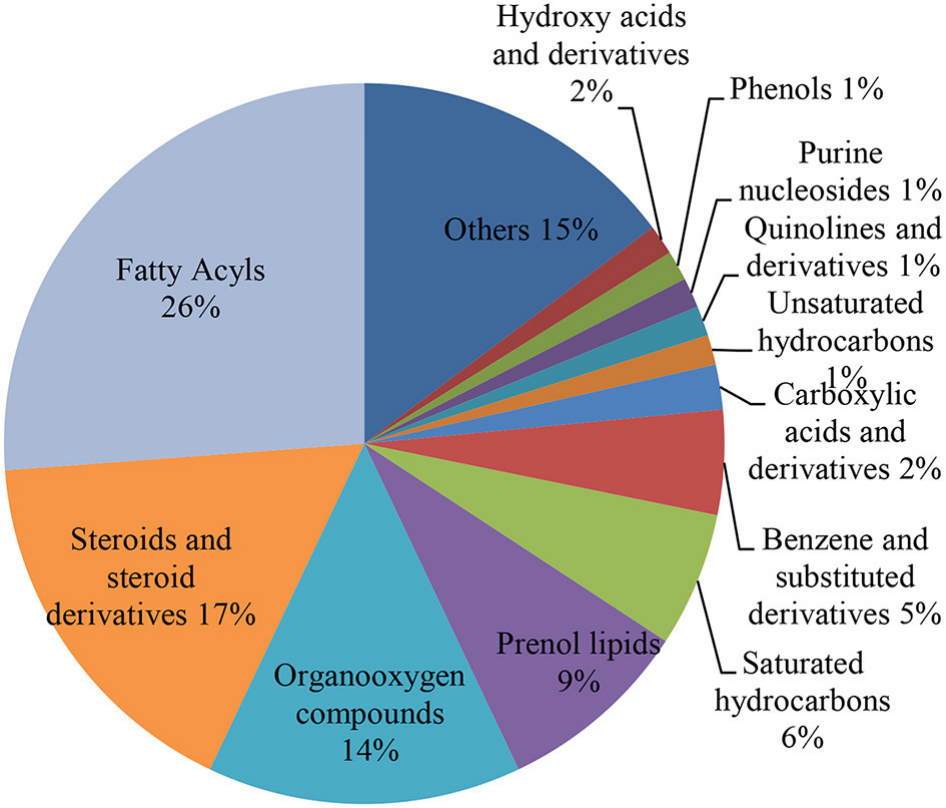
Pie chart illustrating the percentage of identified metabolites for each metabolite class. The various colors in each part of pie chart indicate various HMDB classifications.

Fatty acids shared 26.0% of the detected metabolites. Other contributions are made by steroids and steroid derivatives (17.0%), organooxygen compounds (14.0%), prenol lipids (9.0%), benzene and substituted derivatives (5.0%), carboxylic acids and derivatives (2.0%), and saturated hydrocarbons (6.0%) (Figure 2). The classes that predominate include fatty acyls (31.1–56.3%), steroids and steroid derivatives (1.80–22.4%), dihydrofurans (8.98–11.6%), prenol lipids (0.66–4.44%), organooxygen compounds (0.12–6.45%), benzene and substituted derivatives (0.53–3.73%), glycerolipids (0.36–2.28%), hydroxy acids and derivatives (0.03–2.70%), and benzothiazoles (1.23–3.17%) (Supplementary Table S1).

Elaidic acid (0.32–21.5%), cis-vaccenic acid (0.10–21.4%), pentadecanoic acid (0.37–0.86%), and oleic acid (0.10–22.3%) were found to be the primary fatty acid components in the five rice varieties. The other fatty acids found in significant concentrations were tetracosanoic acid (0.46–0.86%), pelargonic acid (0.06–0.07%), and methyl stearic acid (0.25–3.19%). While a higher amount of palmitic acid was found in Chithiraikar (10.23%), capric acid (1.08%), and panaxydol (0.03%) were found only in Chinnar and Kichili samba, respectively.

Among the 25 steroid compounds, digitoxin (0.03–0.13%), cholestan-3-ol (0.01–0.33%), campesterol (0.01–1.13%), alpha-sitosterol (2.13–6.32%), and (22E, 24R)-stigmasta-4, 22-diene-3,6-dione (0.13–4.57%) were commonly detected in all the rice varieties. Predominate and specific metabolites identified in each rice variety are as follows: 16b-hydroxyestradiol, desmosterol, glycocholic acid in Thooyamalli; 9,19-cyclolanost-24-en-3-ol, (3á)-, calcitriol, á-D-mannofuranoside, 2,3:5,6-di-O-ethylboranediyl-1-O-(stigmasta-5, 22-dien-3-yl)-, and ergosta-5,22-dien-3-ol, acetate, (3á,22E) in Karunguruvai; cycloartenol in Kichili samba; and vitamin D3 in Kichili samba and Chithiraikar.

D-fructose, D-glucose, L-galactose, melezitose, melibiose, and nonanal were the commonly found carbohydrates and their conjugate-based organooxygen compounds (21 metabolites) among the five rice varieties. Out of 13 prenol lipids, cis-sesquisabinene hydrate, cymbopogonol, dehydroabietic acid, gamma-tocopherol, geranyl-PP, longispinogenin, Petasalbin, rhodopin, spirost-8-en-11-one, 3-hydroxy-, (3á,5à,14á,20á,22á,25R)-, and sugiol are the unique ones. All the saturated hydrocarbons (9) found in rice are alkanes. N-dodecane, 2-methylhexacosane, isopentacosane, and 3-methyltetradecane were found only in Chinnar, Thooyamalli, Karunguruvai, and Chithiraikar, respectively. However, a major olefin compound, azulene (0.38%), was accumulated only in Kichili samba. Though the benzene and substituted derivative classes were not detected in Kichili samba and Thooyamalli, the predominant of benzene compound 2,4-di-tert-butylphenol was detected among the other three varieties. Ascaridole was the only dioxane class present in Chinnar and Kichili samba. Among the two phenolic metabolites, *viz*., o-cresol and 2-methoxy-4-vinylphenol, the former was only recorded in Karunguruvai.

**Figure 3:**
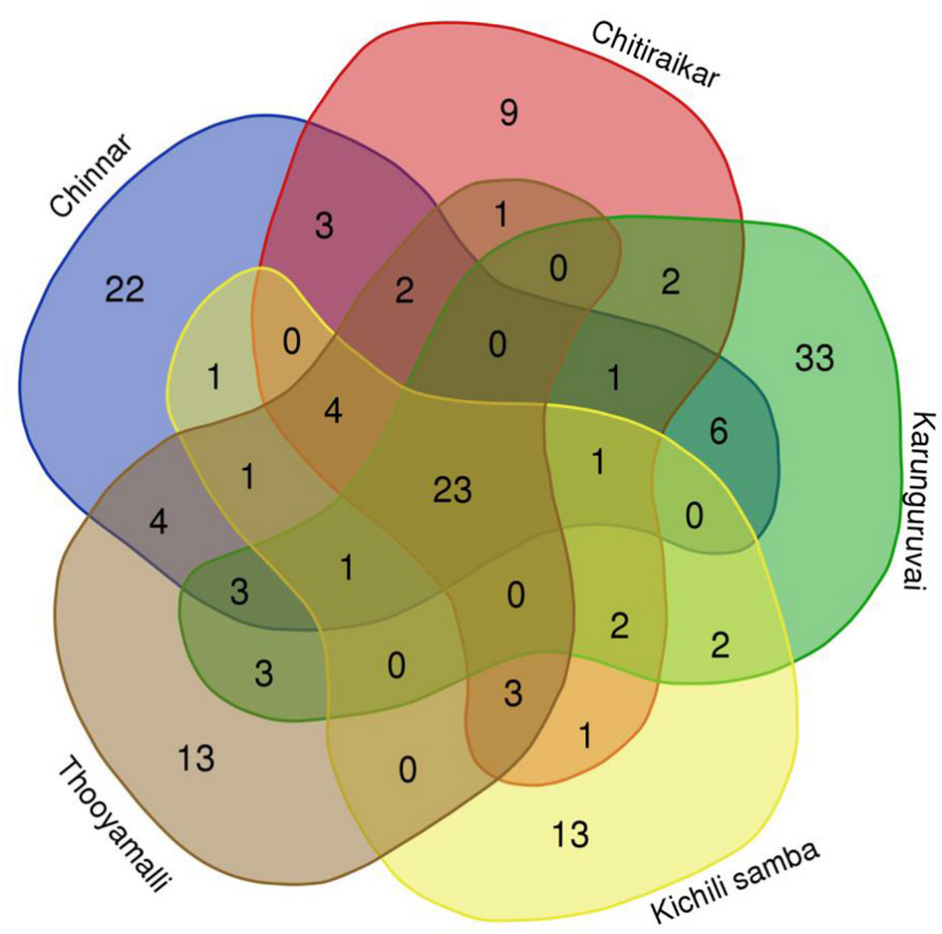
Venn diagram showing number of the commonly shared and differing metabolites among the five traditional rice varieties.

The number of metabolites in traditional rice grain metabolomes commonly present or differing among the five varieties was studied and is shown using a Venn diagram (Figure 3 and Supplementary Table S2). All five rice varieties showed 23 metabolites in common, and each variety had its own special metabolites. Out of the 149 metabolites, 23 (7 upregulated and 6 downregulated) were commonly found among the five rice varieties belonging to Aspidosperma tan-type alkaloids, dihydrofurans, fatty acyls, hydroxy acids and derivatives, organooxygen compounds, prenol lipids, saturated hydrocarbons, and steroids and steroid derivatives (Supplementary Figure S6). The remaining nine compounds were either increased or decreased in one of the rice varieties. For instance, (22E, 24R)-stigmasta-4,22-diene-3,6-dione was decreased in Chinnar, Karunguruvai, and Thooyamalli. However, the concentration of 1,25-dihydroxyvitamin D3-26,23-lactone was reduced in Kichili samba. Metabolomes such as 14-methylicosanoic acid, heptadecanoic acid, pentadecanoic acid, and tetradecanoic acid declined in Karunguruvai compared to other rice samples. A Venn diagram showed that 33 metabolites were specific to Karunguruvai, 22 to Chinnar, 9 to Chithiraikar, and each 13 to Kichili samba and Thooyamalli. It was found that 13 fatty acids and 4 steroids were common to all five varieties.

### 3.1. Univariate analysis (ANOVA)

**Figure 4:**
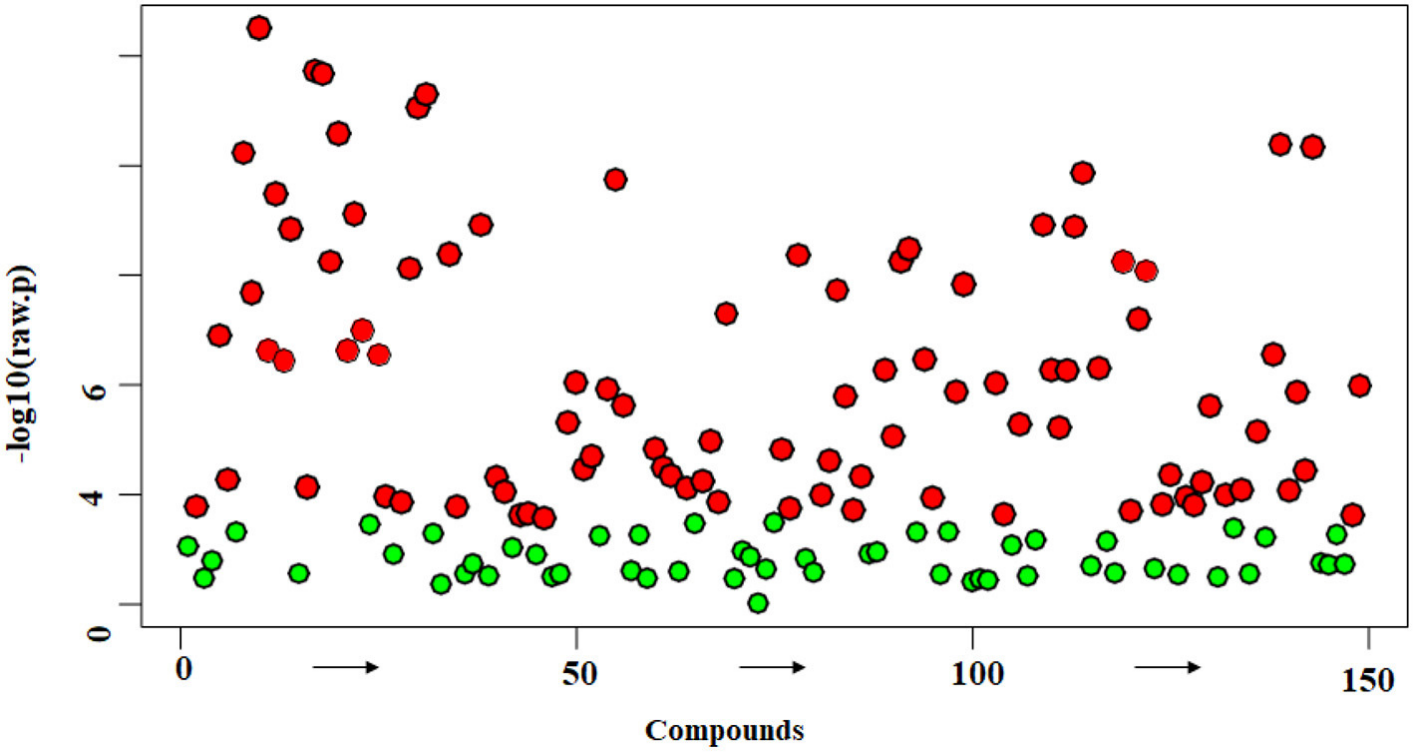
Analysis of variance (ANOVA) plot showing significantly (*P* < 0.05) detected metabolites in traditional varieties. Each red dot represents one metabolite with significance, while the green dot represents without statistical significance. The detailed metabolite identities are given in Supplementary Table S2.

Both univariate and multivariate statistical analyses were carried out to assess the changes in metabolic profiles among the rice varieties. The one-way analysis of variance (ANOVA) statistical test illustrated how the identified metabolites varied among the five rice varieties (Figure 4). While the red color indicates the significant metabolites among the rice varieties, the green color indicates the metabolites of non-significance. Out of the 149 metabolites, 94 distinct metabolites were found to vary significantly (*P* ≤ 0.05) across all the varieties. The precise identities and *P*-value of the metabolites are shown in Supplementary Table S3.

### 3.2. Principal component analysis

**Figure 5:**
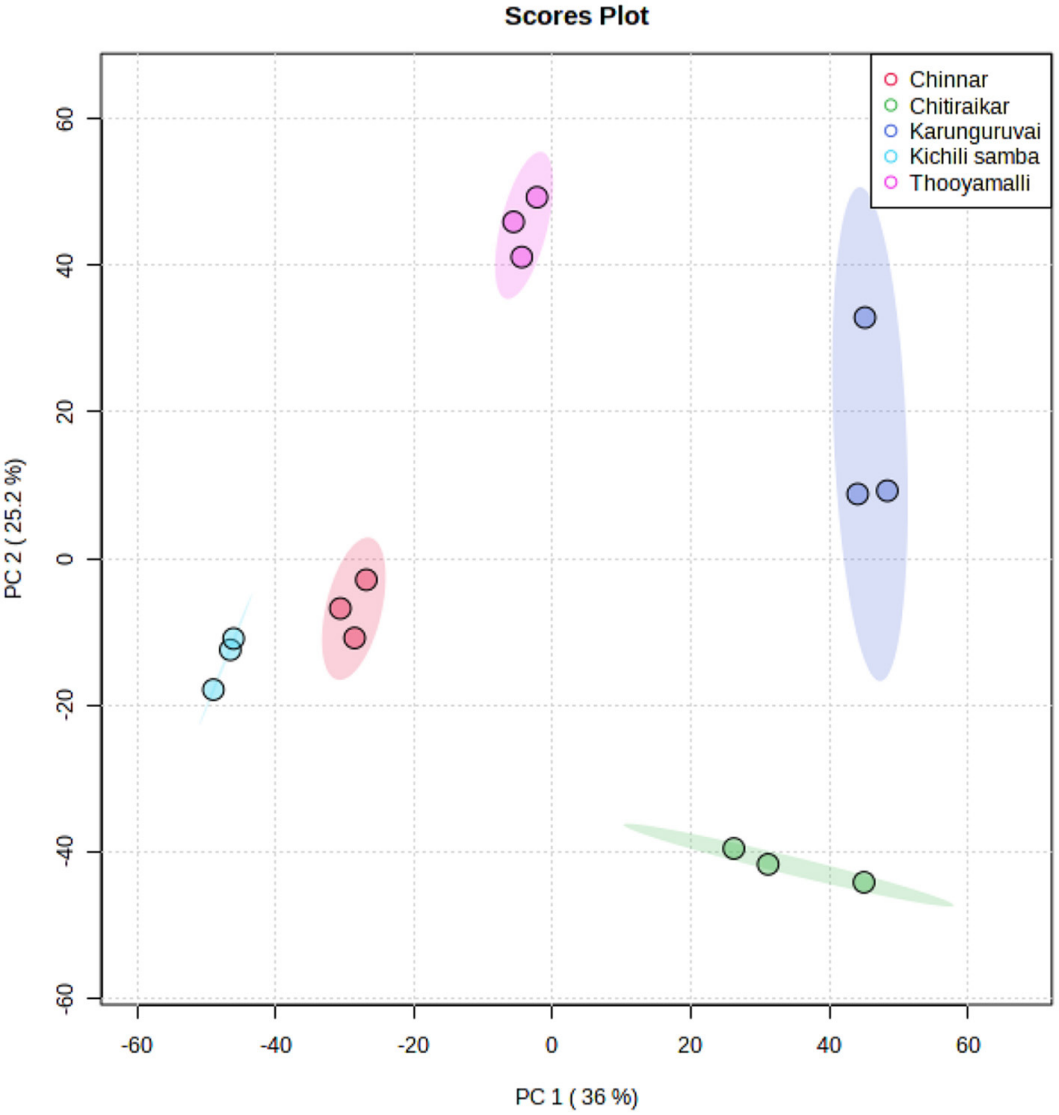
Score plot of PCA.

The processed grain metabolite list was subjected to chemometric analysis techniques such as principal components analysis (PCA), partial least squares-discriminant analysis (PLS-DA), and hierarchical clustering analysis (HCA) to reduce the data dimensionality and enhance data interpretability. All the identified 149 metabolites were subjected to PCA analysis and were reduced to five major principal components (PCs) using the normalized data that indicates major differences between the metabolite data. The PCs with eigenvalues >1 were retained in the study. Figure 5 and Supplementary Figure S6A show the score plots for the five rice varieties. According to the PCA score graph, the metabolite compositions among the five rice varieties differed statistically and significantly from one another. In the PCA score plot, Kichili samba and Chinnar are closely located, which shows a high degree of metabolite similarity among them. The chosen PCs between the score plots indicated that the metabolite loadings of Kichili samba and Chinnar were on the negative side and the others remained on the positive side.

The extracted five PCs explained 96% of the total variance as depicted in the scree plot (Supplementary Figure S6B and Supplementary Table S4). The 36% of variance was explained by PC1 and was correlated with dimethylcycloergostenol, decenal, methyl decenol, dimethoxynonane, dihydroxyvitamin lactone, methylicosanoic acid, methylnonadecanoic acid, methylenecycloartanol, hydroxymethyl furancarboxaldehyde, elaidic acid, heptadecanoic acid, oleic acid, palmitic acid, and tetracosanoic acid. Similarly, the PC2 (25.2% variance) was associated with dioxolane, hydroxyestradiol, di-tert-butylphenol, benzothiazole, and dodecanoic acid in addition to the PC1 metabolites. Similarly, 16.8% of the variance contributed to PC3 was correlated with cis-vaccenic acid, eicosenoic acid, methoxy vinylphenol, hydroxydodecanoic acid, ascorbic acid, and pentadecanoic acid. The PC4 with 14.6% variance was attributed by hexadecanethiol, hexadecane, and octadecane, and PC5 (3.4%) was majorly highlighted by methylenecycloartanol, methoxy vinylphenol, and hydroxymethyl furancarboxaldehyde. The PC analysis showed that the rice varieties differed significantly in terms of both individuals and combinations (Supplementary Figure S6C).

### 3.3. Pathway topology analysis and pathway impacts

**Figure 6:**
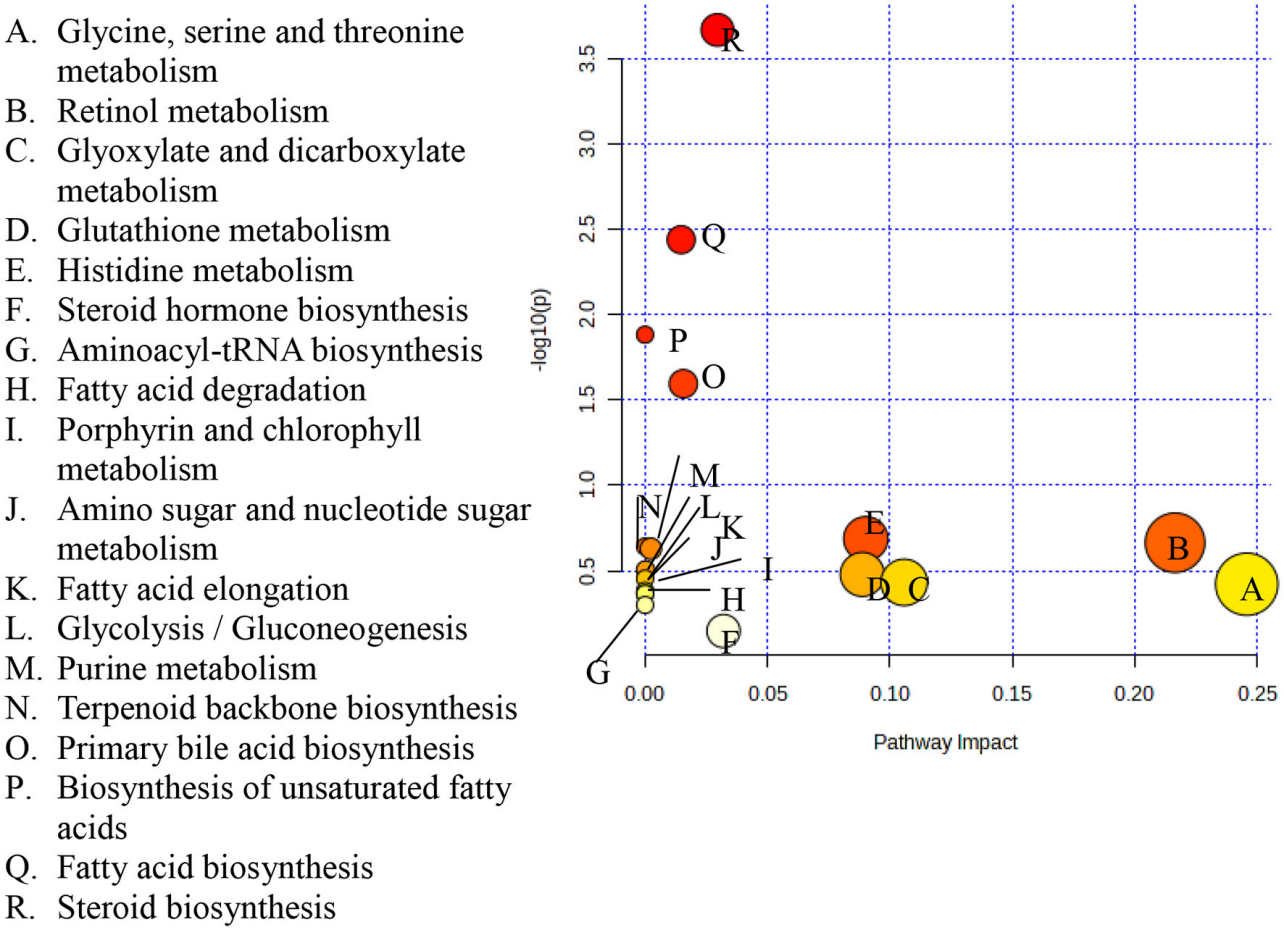
Metabolic pathways identified among the five traditional rice varieties. Each bubble in the plot represents a metabolic pathway whose abscissa and bubble size jointly indicate the magnitude of the impact factors of the pathway in the topological analysis. A larger bubble size indicates a larger impact factor.

A pathway topology analysis was carried out to identify the major metabolic pathways in rice varieties, and the outcome depicting the pathway of distinct metabolites from the five traditional rice varieties is presented as a bubble chart in Figure 6. Approximately 18 important metabolic pathways were identified by pathway topology and impact values (cumulative percentage of the metabolite nodes). Four significant metabolic pathways with false discovery rate (FDR) values ≤0.05 were found when the Kyoto Encyclopedia of Genes and Genomes (KEGG) database was used to map the pathways of significant metabolites. The steroid biosynthesis pathway had the highest log (*p*) value of 3.6682 and was followed by the fatty acid biosynthesis pathway, which had a value of 2.4391. Furthermore, the unsaturated fatty acid biosynthesis pathway with a value of 1.8831 and primary bile acid biosynthesis (1.5955) were mapped (Supplementary Table S5 and Figure 5).

The pathways that have greater impact values are glyoxylate and dicarboxylate metabolism, glycine, serine and threonine metabolism, steroid biosynthesis, fatty acid biosynthesis, primary bile acid biosynthesis, histidine metabolism, retinol metabolism, glutathione metabolism, and steroid hormone biosynthesis.

### 3.4. Hierarchical clustering and heatmap analysis

The differential metabolites found in the present investigation demonstrated numerous biologically identical or complimentary roles, and they were positively or negatively controlled by the same metabolic pathway. In order to further investigate this metabolite dataset, an agglomerative hierarchical clustering analysis model was built based on the metabolites to assess the similarities in the detection of inter-group variance in metabolite features of the grain samples. The differential metabolites were grouped using a complete-linkage method and shown as thermograms after the Euclidean distance matrix was calculated for the quantitative values of each group of comparisons. A dendrogram was used to display the cluster memberships of the traditional rice samples along with heatmap visualization.

**Figure 7:**
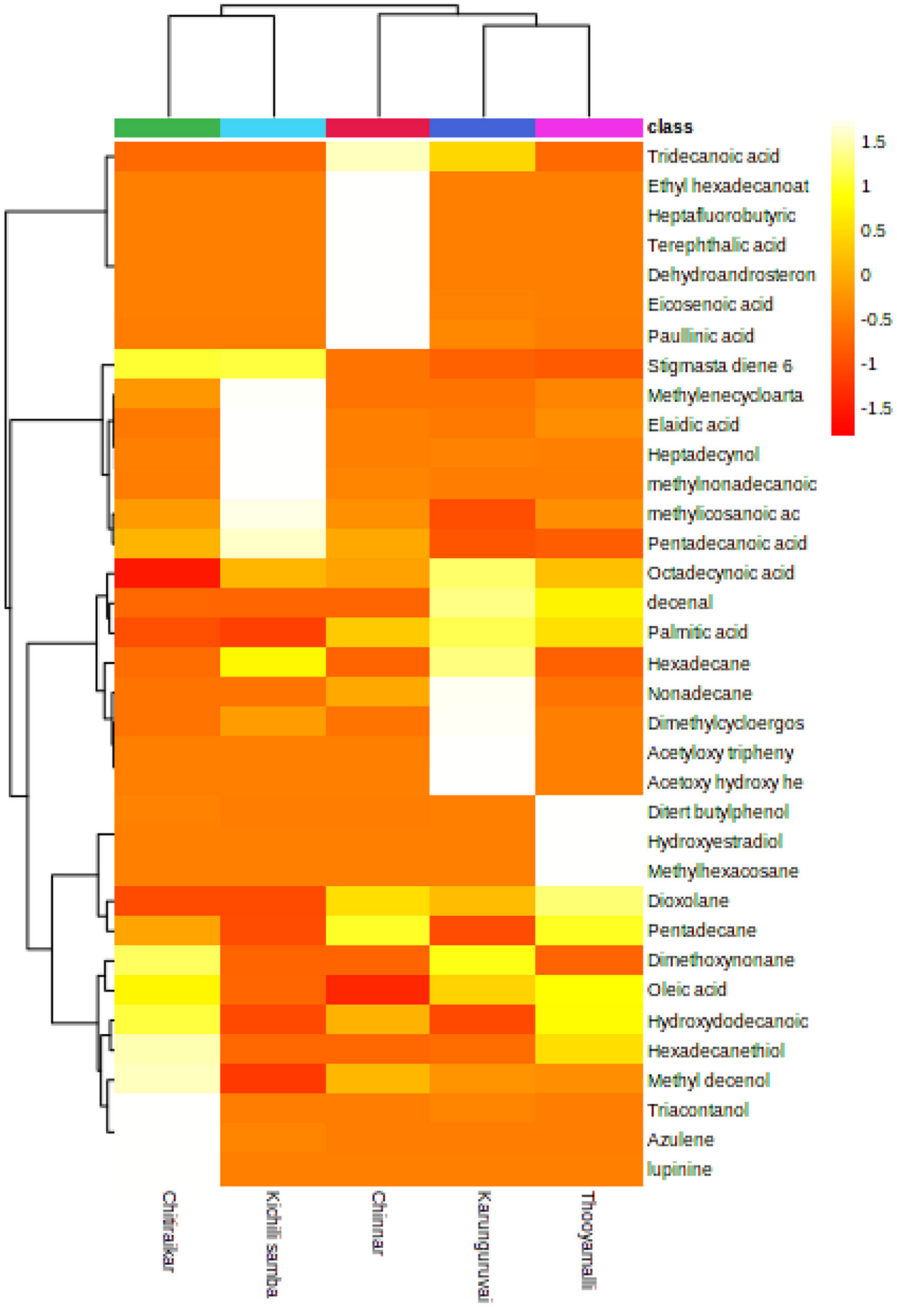
Heatmap representing the changes in soluble metabolites related to five traditional rice varieties which were drawn in MetaboAnalyst 5.0 platform. Each rice sample is shown as a single column in the heatmap, and each metabolite is shown as a single row. Different shades represent metabolite accumulation, whereas red represents the trend of decrease and yellow represents a rising trend (color key scale on the right of heatmap).

The pattern of the top 35 metabolite distributions among the samples is visualized as a heatmap (Figure 7). Metabolites are shown in decreasing order of content by the color gradation from red to yellow. More than 50% of the highlighted metabolites are present in the samples with high relative abundances. While cluster 1 consisted of two varieties, namely Chithiraikar and Kichili samba, cluster 2 was found to consist of Chinnar, Karunguruvai, and Thooyamalli. These two clusters are distributed equally with similar distances; however, the varieties in cluster 1 are chemically similar to each other.

### 3.5. Variable importance in projection

Unsupervised models such as PCA analysis are used to separate the sample based on the variations in factors, whereas supervised analysis explains how far the samples can be discriminated by a particular factor. Partial least squares-discriminant analysis (PLS-DA) is a supervised classification of multivariate analysis that identifies sample variation through dimension reduction and correlation among the variables and is a projection method.

**Figure 8:**
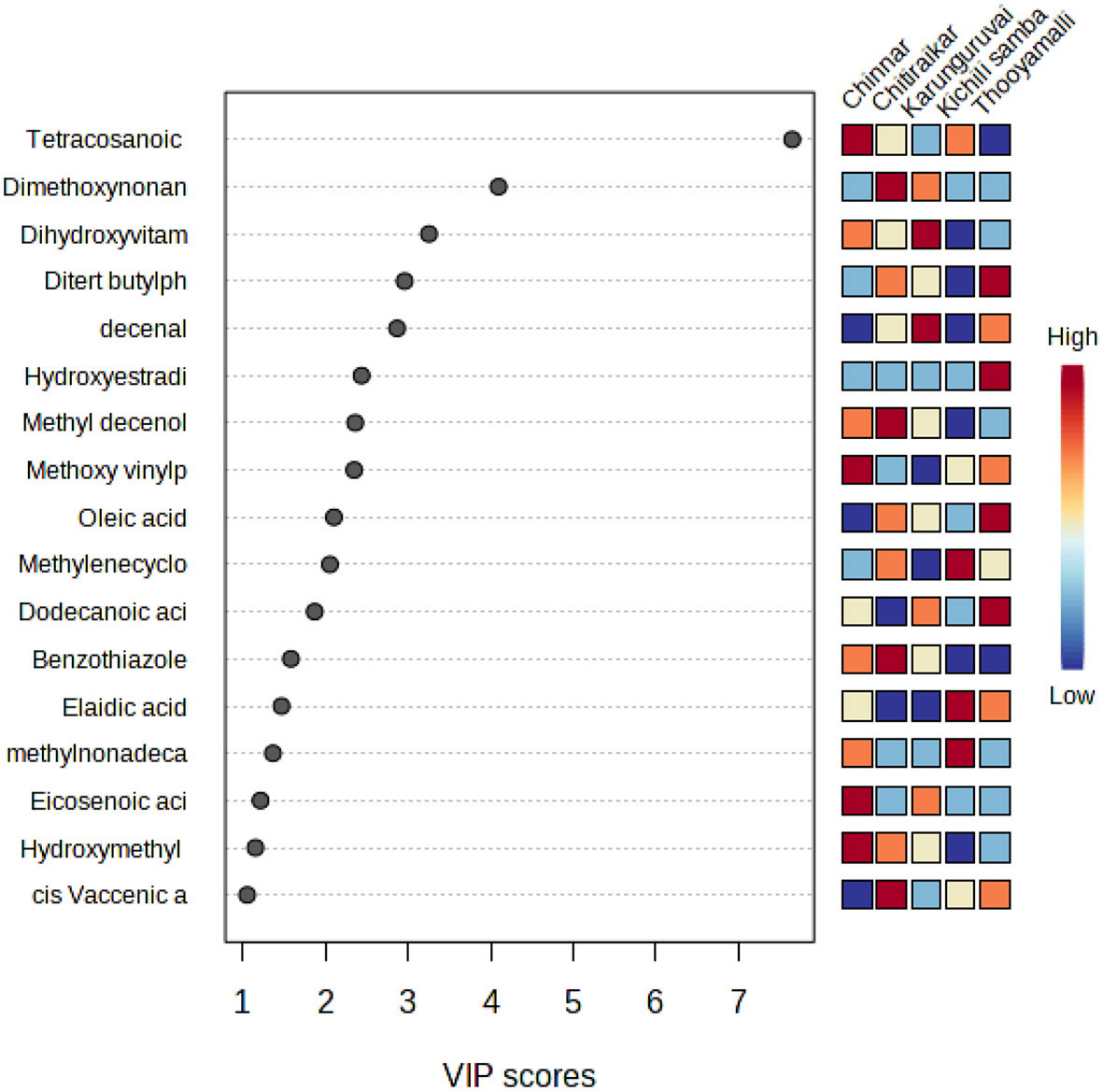
VIP score plot showing the top 17 (score >1) most important metabolite features identified by PLS-DA. The statistical analysis was carried out with the same dataset as in Figure 5.

To assess the compounds responsible for the major differences between the traditional rice varieties, a variable importance in projection (VIP) plot by PLS-DA was employed. Fold changes of >2 or 0.5 were fixed as screening thresholds in combination with PLS-DA model analysis (VIP > 1.0) to identify significantly different metabolites among the five rice varieties (Supplementary Table S6). Seventeen compounds that had a VIP score higher than 1.0 are Tetracosanoic acid, dimethoxynonane, dihydroxyvitamin lactone, di-tert-butylphenol, decenal, hydroxyestradiol, methyl decenol, methoxy vinylphenol, oleic acid, methylenecycloartanol, dodecanoic acid, benzothiazole, elaidic acid, methylnonadecanoic acid, eicosenoic acid, hydroxymethyl furancarboxaldehyde, and cis-vaccenic acid (Figure 8).

### 3.6. Correlation

**Figure 9:**
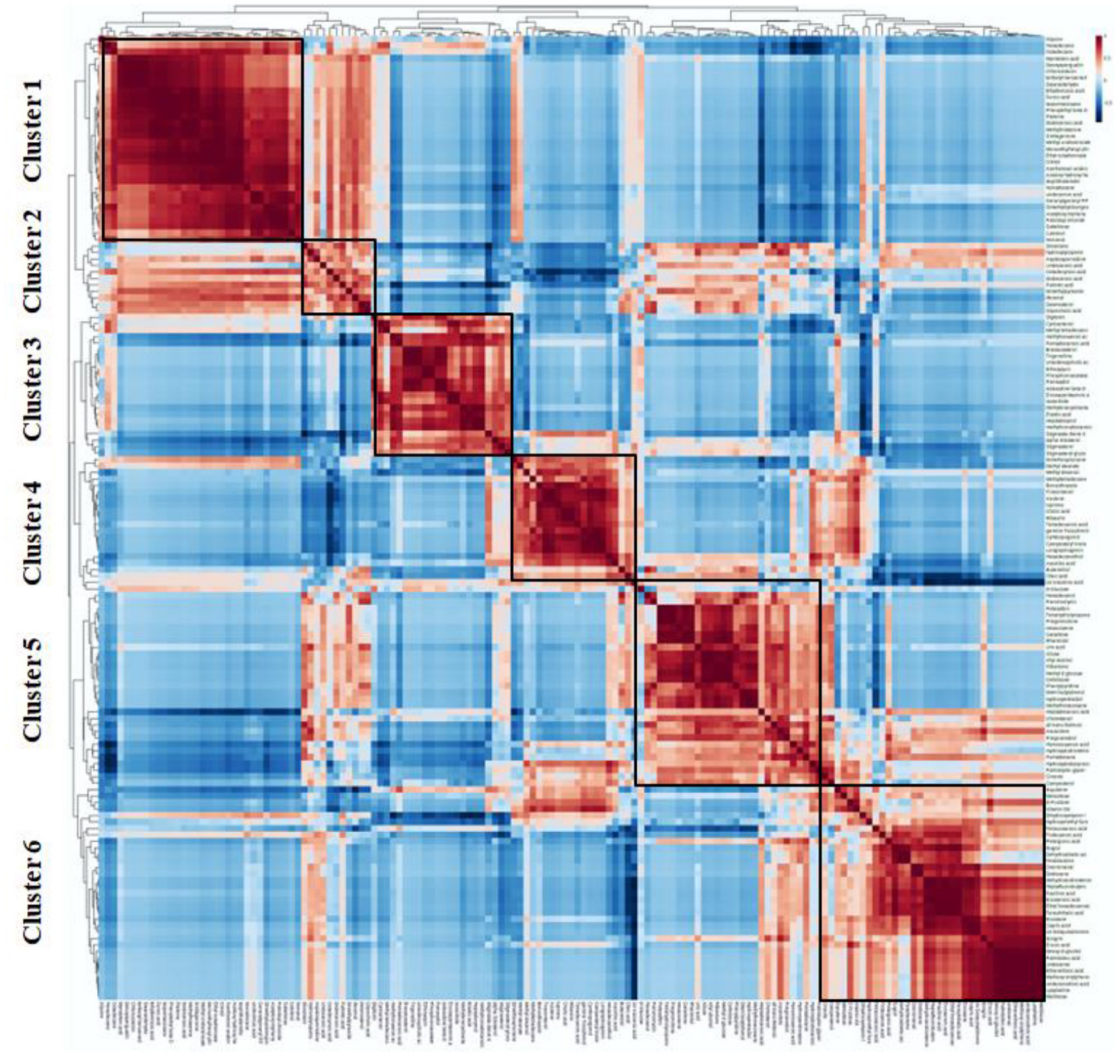
Correlation matrix and cluster analysis of data for 149 metabolites of traditional rice varieties.

We conducted Person's correlation coefficient analysis of the five rice varieties to better comprehend the relationship between metabolite composition and discovered 2,438 significant correlation coefficients (*p* < 0.01, *r*^2^ ≥ 0.49) in the study, which included 1,976 and 463 positive and negative significant correlations, respectively (Figure 9). The findings revealed that certain sugars, fatty acids, and amino acids, including proline, norleucine, and phenylalanine, had the greatest concentration fluctuations. These sugars included fructose, glucopyranose, and raffinose. Correlation clustering divided the 149 metabolites into six clusters that took part in closely associated metabolic pathways. Fatty acids, sugars, hydrocarbons, and cresol were placed in cluster 1. Cluster 2 contained 11 compounds, *viz*, fatty acids, steroids, sugar, and alkaloids, which are positively linked. Of the 22 compounds found in cluster 3, 10 were steroids, 8 were fatty acids, isosorbide, phosphonoacetate, and ethoxyquin. Cluster 4 claimed 20 metabolic features, and the majority of them were fatty acids and prenols. Clusters 5 and 6 were dominated by primary metabolites with few secondary metabolites.

## 4. Discussion

The present investigation was carried out to elucidate the complex grain metabolome of the well-known nutritious and medicinal rice varieties of the Cauvery deltaic region of Tamil Nadu. The primary and secondary metabolites are abundantly found in rice grains. The synthesis of macromolecules that are important for yield and quality, such as starch, is facilitated by primary metabolites. Rice has the capacity to amass an extensive array of secondary metabolites, encompassing phenolic acids, flavonoids, terpenoids, steroids, and alkaloids. These molecules showcase attributes advantageous to humans (Table 3), such as cytotoxic, antitumor, anti-inflammatory, antioxidant, and neuroprotective qualities. As an illustration, numerous antioxidant activities are attributed to elements such as phenolic acids, flavonoids, tocopherols, tocotrienols, γ-oryzanol, and phytic acid found within rice (43).

**Table 3:** Detailed information about important metabolites and their corresponding biological significance.

**Sl. no**.	**Important metabolites**	**Biological significance**	**References**
1.	Azulene	Anti-inflammatory activity	Guarrera et al. (24)
2.	*p*-Cresol	Anticancer activity	Hinai et al. (25)
3.	Palmitic acid	Increases the risk of cardiovascular diseases	Fattore and Fanelli (26)
4.	Elaidic acid	Anticancer	Bergman et al. (27)
5.	Oleic acid	Reduces cardiovascular risk by reducing blood cholesterol	Priore et al. (28)
6.	Squalene	Reduces wrinkles and decreases UV-induced DNA damage in human skin	Cho et al. (29)
7.	Stearic acid	Reduces human breast cancer	Khan et al. (30)
8.	2,4-Di-*tert*-butyl phenol	Antioxidant Antifungal	Varsha et al. (31)
9.	Hydroxy lycopene	Anticancer, antidiabetic, cardioprotective, antioxidative, anti-inflammatory, hepatoprotective, neuroprotective	Imran et al. (32)
10.	Paromomycin	Antibiotic against visceral leishmaniasis	Sundar et al. (22)
11.	Sugiol	Antimicrobial, antioxidant, anti-inflammatory, anticancer, antiviral, and antitumor	Bajpai et al. (21)
12.	Campesterol; Brassicasterol; Sitosterol; Stigmasterol	Reduces blood cholesterol levels: total cholesterol and LDL cholesterol and inhibits cholesterol absorption	Sanclemente et al. (33)
13.	Eicosapentaenoic Acid	Anti-inflammatory process!!!!kannan!!!! Proper fetal brain and retina development	Smith et al. (34) Ramakrishnan et al. (35)
14.	Cymbopogonol	Antimicrobial activity	Abdelsalam et al. (36)
15.	Ursodeoxycholic acid	Beneficial effects in cholestatic disease	Ikegami and Matsuzaki (37)
16.	Cycloartenol	Lowers postprandial and hyperglycemia	Okahara et al. (38)
17.	Trigonelline	Cancer chemopreventive	Zhou et al. (39)
18.	Aldehydes, Nonanal, and Decanal	Olfactory simulation effect	Kim et al. (40)
19.	Glycine	Anti-inflammatory and immunomodulatory	Zhong et al. (41)
20.	Undecanoic and Capric acid	Immune function	Roopashree et al. (42)

Additionally, several secondary metabolites have dietary purposes. For instance, the outer layer of rice grain has a distinctive profile of phytochemicals with therapeutic and dietary benefits for human health. A few researchers have attempted to quantify the biochemical constituents in the traditional rice grain varieties and the metabolomics of one or two in comparison with modern varieties (11, 12). However, no comprehensive study has examined the metabolite profiling of the nutritionally and therapeutically rich traditional rice varieties belonging to the Cauvery delta region of southern India. After COVID-19, traditional rice has drawn increased interest from nutritionists, consumers, and health advisors because of its higher significance in terms of its biological activity, nutritional content, and significant impact on human health. They are more concentrated in fiber, iron, calcium, vitamins, minerals, and other bioactive compounds than modern and hybrid rice varieties. Furthermore, Indian traditional rice helps increase the milk secretion of lactating mothers and is used to cure a variety of illnesses, including rheumatism, leukorrhea, gastrointestinal problems, and skin conditions (12).

In addition to being significant energy sources, carbohydrates serve as the building blocks for the production of starch and maintain metabolic homeostasis, as well as a number of other processes, including the pathology and physiology of the large intestine, dental health, and the prevention of the onset of chronic non-communicable diseases. These mechanisms are contingent upon the specific carbohydrates ingested, their quantities, and their equilibrium with other essential nutrients. The current study confirms the presence of monosaccharides, disaccharides, oligosaccharides, aldehydes, alcohols, and polyols in all the traditional rice varieties. Paromomycin, an antibiotic belonging to the aminoglycoside class found only in Thooyamalli, has demonstrated dose-dependent effectiveness in addressing visceral leishmaniasis (22). The compound 9-undecenal, identified within Chinnar, has been established to exhibit odorant receptor properties, as evidenced by the research of Trimmer et al. (44). Other organooxygen compounds found in this investigation are sinigrin (antiproliferative activity) (45) and nonanal (olfactory simulation effect) (40).

Fatty acids are a group of lipids that play a crucial structural, functional, and biological role in addition to serving as a significant energy source. Most of these fatty acids can be produced by the human body, with the exception of important polyunsaturated fatty acids (PUFAs), *viz*., omega-3, omega-6, and omega-9, which are found in traditional rice varieties. Omega-3 and omega-6 fatty acids can also lower blood cholesterol levels and the risk of cardiovascular diseases (23). Eicosapentaenoic acid (EPA) is one of the significant omega-3 fatty acids present only in Kichili samba. It plays an essential role in the anti-inflammatory process (34) and proper fetal brain and retina development (35). EPA enhances plaque stability, lessens endothelial activation, and increases vascular permeability, which lowers the risk of suffering a cardiovascular issue (46). By influencing important blood glucose metabolism enzymes, palmitoleic acid lowers insulin resistance in diabetics and controls insulin secretion in patients (47). According to earlier research findings (48), the two fatty acids, namely oleic acid and linoleic acid, which make up 75% of the total unsaturated fats in rice bran oil, are primarily responsible for decreasing cholesterol levels. Oleic acid is a monounsaturated fatty acid that can reduce blood cholesterol levels, improve cell membrane permeability, and prevent myocardial block and arteriosclerosis (49). Our study agrees with the literature that the *Garudan Samba* possesses 9,12-octadecadienoic acid (Z, Z), which is responsible for anti-arthritic, 5-alpha-reductase inhibiting, hypocholesterolemic, hepatoprotective, anti-androgenic, anti-coronary, and anti-histaminic effects (50).

Rice terpenoids include monoterpenoids, sesquiterpenoids, diterpenoids, and triterpenoids. 1,4-Cineole is one of the monoterpenoids that has anti-inflammatory activity (51). Squalene, a triterpenoid molecule with pharmacological significance that exhibits anti-inflammatory, immunostimulant, anticancer, and antioxidant properties (52), is also present in all five rice varieties. Squalene's main role is to shield the surface of human skin from lipid peroxidation brought on by exposure to UV and other ionizing radiation (53). Sesquiterpenoids are another group of secondary metabolites that influence the rice aroma (43). To the best of our knowledge, this is the first time that gamma-tocopherol has been reported in the grains of Chithiraikar which is a quinone and hydroquinone lipid-based metabolite. This has an antioxidant function (54), encourages the healing of plasma membranes (55), enhances cardiovascular performance (56), and reduces the expansion of cancer cells. Our study corroborates previous findings, which also detected the presence of alpha-tocopherol in Mappillai samba (11). Sugiol, a diterpenoid present in Chinnar, has the potential to function as an antimicrobial, antioxidant, anti-inflammatory, anti-carcinoma, antiviral, and cardiovascular agent (21).

In the course of this investigation, we identified two previously undiscovered carotenoids, namely, 7,7′,8,8′-tetrahydrolycopene and 1-hydroxylycopene, present in all five rice varieties. These carotenoids are renowned antioxidants known for safeguarding DNA, proteins, and lipids against oxidative damage (32). All-trans-retinoic acid (ATRA) is a biologically active form of vitamin A and serves as a crucial signaling molecule in various physiological processes. ATRA assumes pivotal roles across a diverse spectrum of biological processes, encompassing embryonic morphogenesis, organogenesis, cell proliferation, differentiation, apoptosis, homeostasis, and their associated disorders (57). Dehydroabietic acid is a prominent example within the aromatic abietane category. Similar to many other diterpenoids, aromatic abietanes are predominantly recognized for their roles as chemical defense agents. This group has been attributed with a range of biological activities, including antimicrobial, antileishmanial, antiplasmodial, antifungal, antitumor, cytotoxic, antiviral, antiulcer, cardiovascular, antioxidant, and anti-inflammatory activities, as documented in existing reports (58).

Inosine is a purine nucleoside found in Karunguruvai and has been under investigation in clinical trials for its potential in treating neurological disorders, including Parkinson's disease (59).

Nine alkanes were present in the studied varieties, which are in agreement with earlier observations by Krishnanunni et al. (19) and Ashokkumar et al. (12, 13) in rice. Eicosane has antimicrobial activity (60) that is found only in Chinnar and Chithiraikar. Tetradecane, hexadecane, and pentadecane are known to possess antifungal and antibacterial effects (61). Azulene is the only unsaturated carbon-based olefin present in Kichili samba, and Chithiraikar possesses antineoplastic, analgesic, antidiabetic, and antiretroviral properties in the treatment of HIV-1 and antifungal properties. Regular intake of Kichili samba builds muscles and increases the immune system (4).

Trigonelline is an alkaloid present in Kichili samba and has been shown to alleviate diabetic auditory neuropathy and platelet aggregation. It also exhibits hypoglycemic, hypolipidemic, neuroprotective, antimigraine, sedative, memory-improving, antibacterial, antiviral, and antitumor properties (39).

Plants' membrane lipid bilayer is made up in large part by plant steroids, also known as phytosterols. They control membrane fluidity, affecting the structure, features, and functions of the membrane (62). In this investigation, all five crucial phytosterols, namely, campesterol, stigmasterol, desmosterol, brassicasterol, and alpha-sitosterol, were observed to be prevalent in all the studied varieties and reported to have a variety of physiological impacts. This contradicts earlier findings concerning conventional rice, as noted by Ashokkumar et al. (12, 13). Campesterol which has antioxidant and hypocholesterolemic properties (19), β-sitosterol is known for its hypocholesterolemic, antisterility, and anticancer properties (63), whereas stigmasterol is being used as a precursor in the synthesis of semi-synthetic progesterone (33). Stigmasterol has additionally been documented to display antihepatotoxic, anti-inflammatory, antioxidant, antiviral, anticancer, and antihypercholesterolemic effects (19). Therefore, consuming pigmented rice as part of a healthy diet is one of the long-term methods to prevent the spread of breast cancer. However, the studied five varieties (white and red) possess all the phytosterols that make them one of the most balanced diets to prevent the metastasis of breast cancer. Traditional pigmented rice cultivars have the ability to sustain glucose homeostasis, making them helpful for the management of diabetes mellitus (64). Consequently, these traditional varieties (unpolished and hand-milled) are popular among local consumers and thought to be the best for diabetes patients due to the presence of significant phytosterols.

The secosteroid vitamin D3 is inversely related to respiratory infection (65) by producing cathelicidin and defensin peptides (62) that have antimicrobial activities against various microorganisms, including bacteria, viruses, and fungi (66). A study conducted by Pinzon et al. (67) found that a vitamin D shortage might be a risk factor for causing viral-related infections. In our study, vitamin D3 was possessed by Chithiraikar and Kichili samba. The popularity of eating these traditional varieties has increased after the COVID-19 pandemic. Hence, we first report here that the presence of seco-steroids in traditional varieties might be the reason for resistance to respiratory-related viral infections. Calcitriol is one of the hormonally active forms of vitamin D present only in Karunguruvai, exerts antiproliferative impacts in various cancers and malignant cells, including prostate cancer, raising the significance of its utilization as an anticancer agent (68). Therefore, this variety can be recommended to people to prevent the formation of malignant cells and also to those having treatment for the same. In spite of this, a detailed study is required to explore the antiproliferative properties of Karunguruvai.

A bile acid derivative, namely glycocholic acid, is present in Karunguruvai, and Thooyamalli is reported to have clinical importance in absorbing fat- and fat-soluble vitamins and reducing the risk of bile acid amidation defects (69). Neurosteroid found only in Thooyamalli is pregnenolone, which exerts anxiety and depression-regulatory mechanisms (70).

Phenolic acids are key auxiliary metabolites generally found in plant-based food varieties, such as grains, organic products, and vegetables, and provide different biological activities, including cancer prevention agents, antimicrobial, antitumor, and mitigating inflammatory issues (71). The main phenolic acids in Cauvery deltaic traditional rice are o-cresol and 2-methoxy-4-vinylphenol. Ethylbenzoic acid was found in the Karunguruvai rice. Ribavirin is a ribonucleoside found only in Chinnar. While exhibiting activity against a wide range of viruses, ribavirin finds its prominent clinical utility in treating respiratory syncytial virus among pediatric patients, along with managing chronic hepatitis C virus (HCV) infection in both the children and adult populations (72).

More than 200 volatile substances, including (E,E)-2,4-decadienal, hexanal, octanal, nonanal, 4-vinylphenol, and 4-vinyl guaiacol, are considered to influence the aroma of rice, with 2-acetyl-1-pyrroline (2-AP) serving as the main volatile aromatic molecule (10). We report the presence of nonanal for the first time in Chithiraikar, Karunguruvai, and Kichili samba.

### 4.1. Pathway topology

The intricate metabolic reactions within living organisms are tightly interconnected and often orchestrated by a multitude of genes and proteins. These components collaboratively construct intricate pathways and networks, intertwining and governing each other's functions. Consequently, this interconnected process culminates in comprehensive modifications to the metabolome, as outlined by Töpfer et al. (73). In our investigation, we meticulously delineated and organized pathways to elucidate the differentially expressed metabolites in the individual traditional varieties.

### 4.2. Heatmap clustering and correlation

To gather pertinent data about the similarities and differences in the metabolite profiles between the grain samples, HCA was built based on the metabolites. The metabolite profiles' diversity was shown through cluster analysis. The clustering of the samples based on the top 35 secondary metabolites demonstrates the analytical parameters' capacity to discriminate in this situation. A single linkage with correlation as a measure of similarity was used to create the dataset. More than 50% of the highlighted metabolites present in the samples have high relative abundances, as demonstrated in Figure 6 (represented in red or yellow).

According to the heatmap-based dendrogram, cluster 1 (Chithiraikar and Kichili samba) is more compact, which implies that the metabolites are comparable with each other within the cluster group. In contrast, cluster 2′s (Chinnar, Karunguruvai, and Thooyamalli) metabolites are comparatively spaced out, thus indicating that they are more chemically diverse than the cluster 1 samples. The metabolite variation within the cluster and between the clusters might be due to the genetic makeup of the individual rice variety and environment (74, 75). Therefore, unequivocally, the HCA results might be affected by genetic differences (76). We also found that this difference was not altered by the growth location, management practice, or harvest time, as they were similar to each other.

The metabolic features present in the clusters were positively correlated with each other. Fatty acids and sugars present in clusters 1 and 2 are positively correlated with each other, except dioxalones, hydroxylycopene, aspidospermidine, and undecanoic acid which are negatively correlated.

### 4.3. Identification of metabolites from variable importance in projection

The variable importance in the projection (VIP >1) and *P*-value of Student's *t*-test (*p* > 0.05) were employed in this experiment to identify the metabolites with differential expression (Figure 7). The 17 differential metabolites with substantial variations were selected from five rice varieties. These metabolites include nine different types of fatty acids, three different kinds of steroids, two benzenes and substituted derivatives, a type of benzothiazole, a type of phenol, and hydroxy acids and derivatives. Out of 17 VIP metabolites, 7 (4 upregulated, 3 downregulated) were common to all the rice varieties. Phenolic acids are viewed as natural antioxidants, having the potential to scavenge free radicals that might increment oxidative pressure (77); hence, rice phenols are always correlated with antioxidant capacity (78). One such phenolic metabolite, 2,4-di-tert-butylphenol (DTBP), present in studied rice varieties has been shown to have a variety of biological properties, including antibacterial (79), antifungal (80), and anticancer activity (31, 81), which was not present in Kichili samba. A methoxy vinylphenol that acts as an anti-cell proliferative agent (82) was not associated with Karunguruvai.

**Figure 10:**
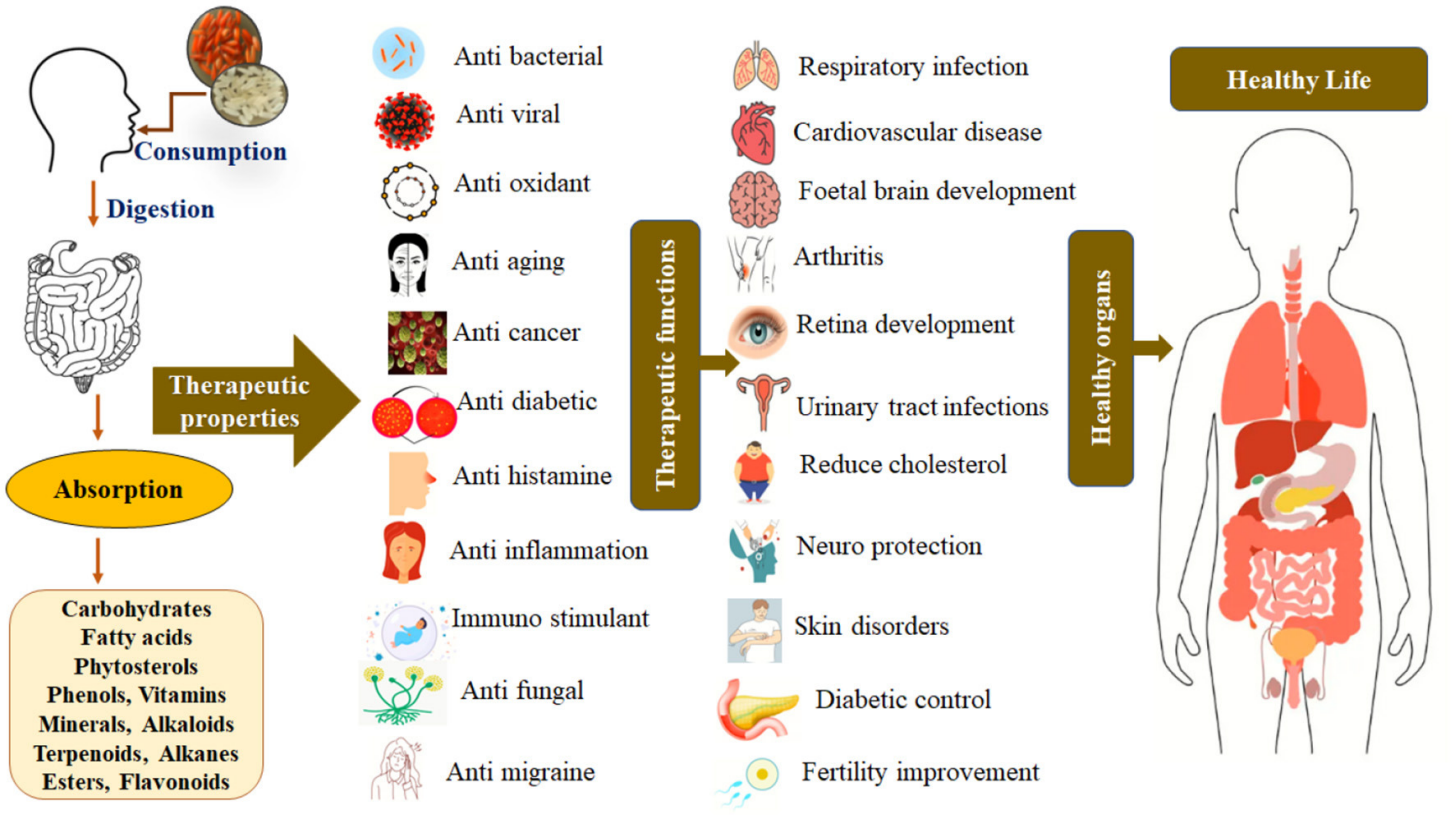
Therapeutic properties of five traditional rice varieties and their functions on human health.

Traditional rice boasts inherent antioxidant content and therapeutic attributes, enabling its integration into an array of functional foods, food additives, nutraceuticals, pharmaceuticals, and cosmetics. Consequently, the study underscores the potential for enhanced health benefits through the regular consumption of traditional rice (Figure 10). To capitalize on this potential, the study recommends an augmentation of production in the processing industry, particularly focusing on the creation of convenient food items such as ready-to-eat, ready-to-cook, and instant foods.

## 5. Conclusion

The present study is the first to provide extensive data on the variation of bioactive metabolomes in the traditional rice varieties (except Kichili samba) of the Cauvery deltaic region, South India. Untargeted metabolomics of five traditional rice varieties showed 149 metabolites under 34 chemical classes exhibiting pharmaceutical and health functions. Further metabolic pathway analysis shows that these traditional rice grain metabolomes have a tremendous role in physiological pathways and hence could be a reference for future breeding programs. The pharmaceutically significant traits of the identified therapeutic/nutraceutical compounds in the grains of these varieties may serve as potential leads for the genetic mapping of these traits, accelerating the development of high-yielding rice varieties with enhanced therapeutic/nutritional qualities. In the current study, it was discovered that the traditional rice cultivars from Tamil Nadu have a wide range of phytochemicals or bioactive compounds, which may help scientists, policymakers, and rice producers to enhance the acreage of cultivation. In Tamil Nadu, roughly more than 400 traditional varieties are available for cultivation. However, only a few of these landraces have undergone a thorough investigation of their grain end-use quality, nutritional characteristics, and possible therapeutic advantages. Therefore, there is an urgent need to validate the science and confirm the traditional knowledge connected to these varieties.

## Data availability statement

The original contributions presented in the study are included in the article/Supplementary material, further inquiries can be directed to the corresponding authors.

## Author contributions

SV: Conceptualization, Data curation, Formal Analysis, Investigation, Methodology, Resources, Software, Writing—original draft, Writing—review and editing. DU: Conceptualization, Data curation, Formal Analysis, Investigation, Methodology, Resources, Software, Writing—original draft, Writing—review and editing. KS: Investigation, Resources, Validation, Writing—review and editing. PJ: Conceptualization, Investigation, Methodology, Resources, Supervision, Visualization, Writing—review and editing. BP: Investigation, Resources, Validation, Writing—review and editing. SS: Investigation, Resources, Validation, Writing—review and editing. SV: Investigation, Resources, Validation, Writing—review and editing. SM: Investigation, Resources, Supervision, Validation, Visualization, Writing—review and editing. NB: Investigation, Resources, Supervision, Validation, Visualization, Writing—review and editing. MR: Validation, Visualization, Writing—review and editing, Investigation, Resources, Supervision. VG: Investigation, Resources, Supervision, Validation, Visualization, Writing—review and editing. ES: Investigation, Resources, Supervision, Validation, Visualization, Writing—review and editing.
