# Supplementary material for: Metabolomic analysis for disclosing nutritional and therapeutic prospective of traditional rice cultivars of Cauvery deltaic region, India
Source: Front Nutr. 2023 Sep 28;10:1254624. doi: 10.3389/fnut.2023.1254624 (PMC10568072; doi:10.3389/fnut.2023.1254624)
Supplement: Supplementary file 1 [file Data_Sheet_1.pdf]

Metabolomic analysis for disclosing nutritional and therapeutic prospective of traditional rice cultivars of Cauvery deltaic region, India

Supplementary figure legends

Figure S1. GC-MS chromatogram of Chinnar

Figure S2. GC-MS chromatogram of Chitiraikar

Figure S3. GC-MS chromatogram of Karunguruvai

Figure S4. GC-MS chromatogram of Kichili samba

Figure S5. GC-MS chromatogram of Thooyamalli

Figure S6. Principle Component Analysis. Fig. S6A shows the 3D of PCA. Fig.S6B shows the scree plot of PCA. Fig.S6C shows the biplot

Figures S7. Commonly observed 23 metabolites among the 5 traditional rice varieties along with the up regulated and down regulated status. Values represented are mean of area intensity obtained from three replications.

Supplementary Table legends

Table S1. Identified 149 metabolites based on their composition

Table S2. Identified 149 metabolites along with retention time for individual variety

Table S3. Venn daigram results of compounds commonly shared by each group and difference among them Identified 149 metabolites along with retention time for individual variety

Table S4. ANOVA for significant metabolites

Table S5. Loadings of the variables in the all principal components.

Table S6. Pathways identified using METABOANALYST in 5 rice varieties

Table S7. VIP scores for metabolites analysed through PLS-DA

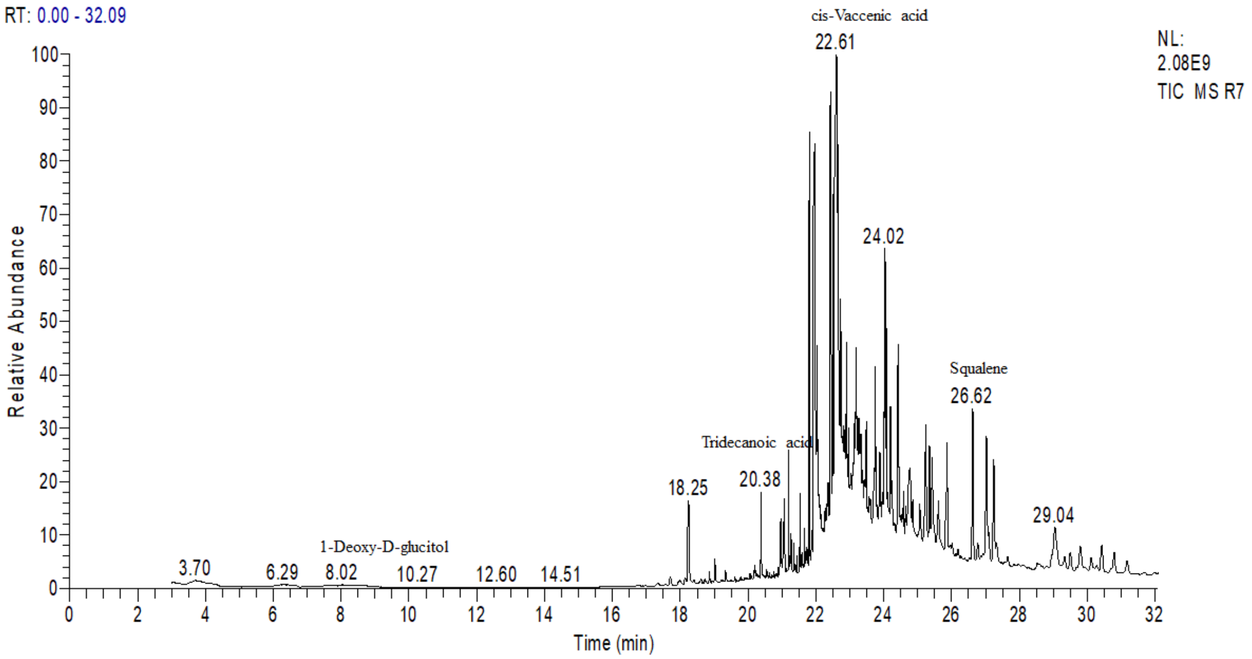

Figure S1. GC-MS chromatogram of Chinnar

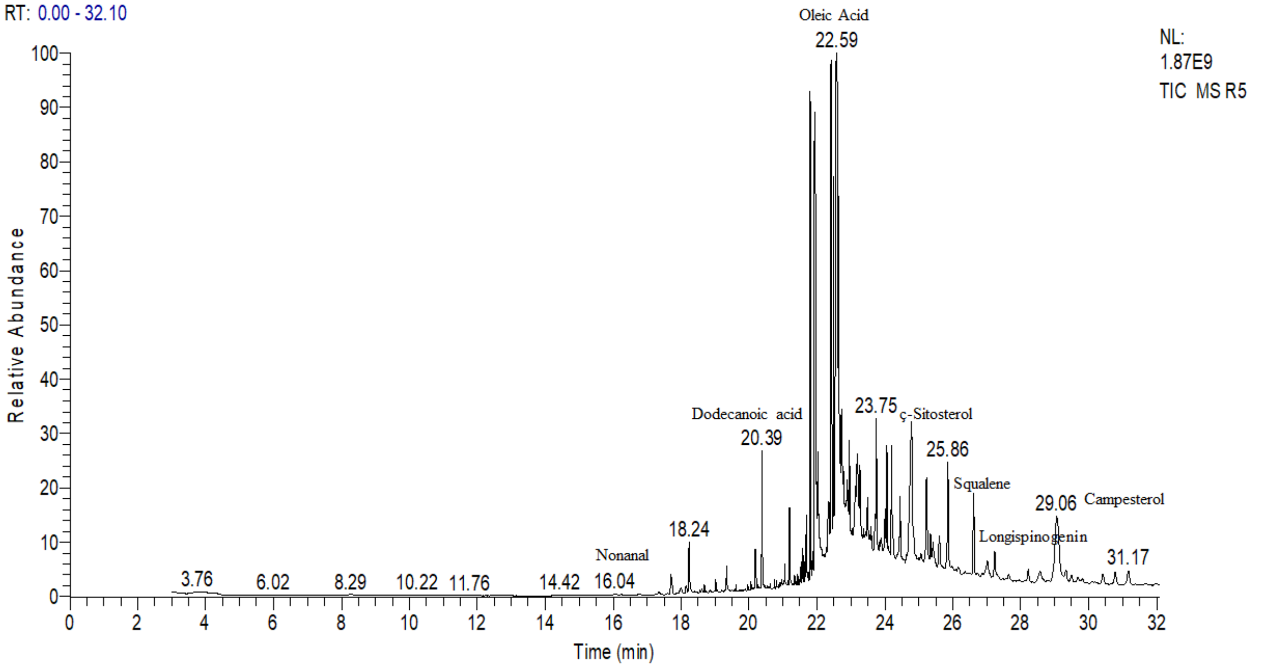

Figure S2. GC-MS chromatogram of Chitiraikar

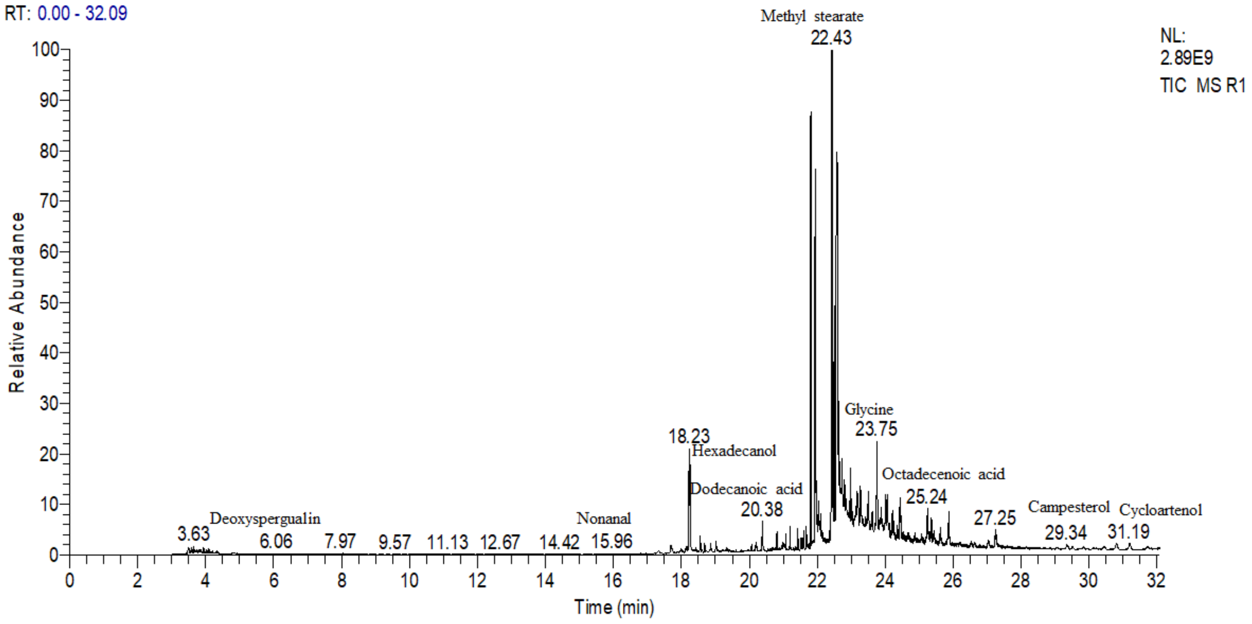

Figure S3. GC-MS chromatogram of Karunguruvai

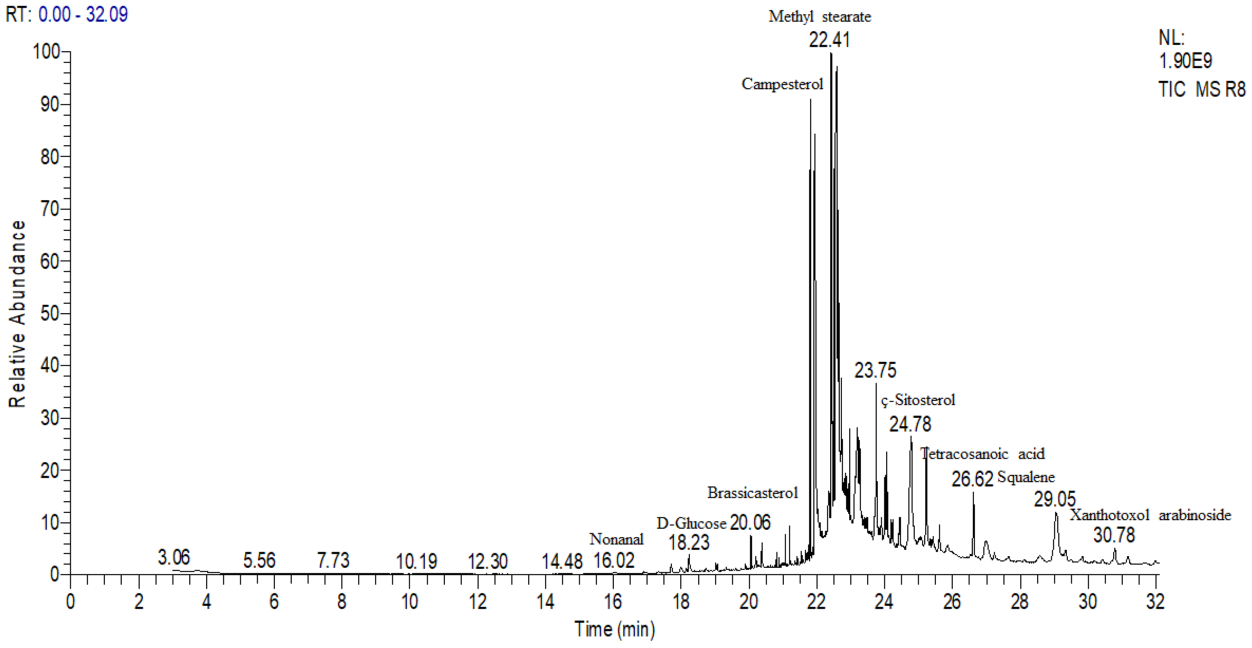

Figure S4. GC-MS chromatogram of Kichili samba

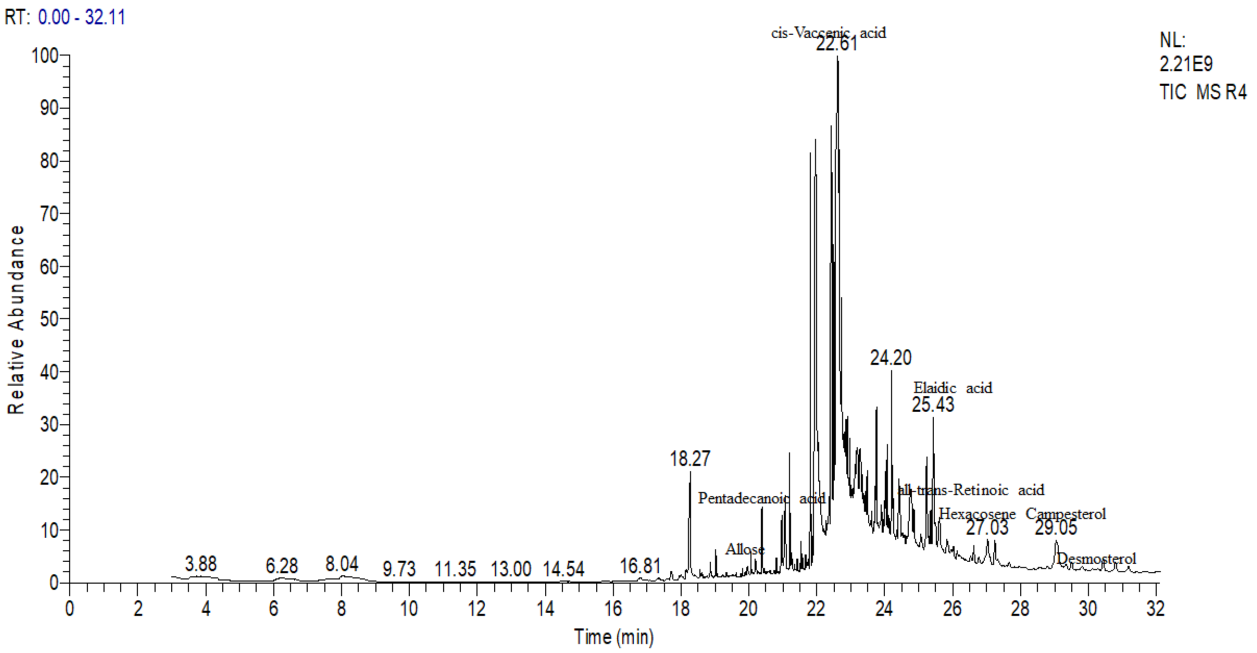

Figure S5. GC-MS chromatogram of Thooyamalli

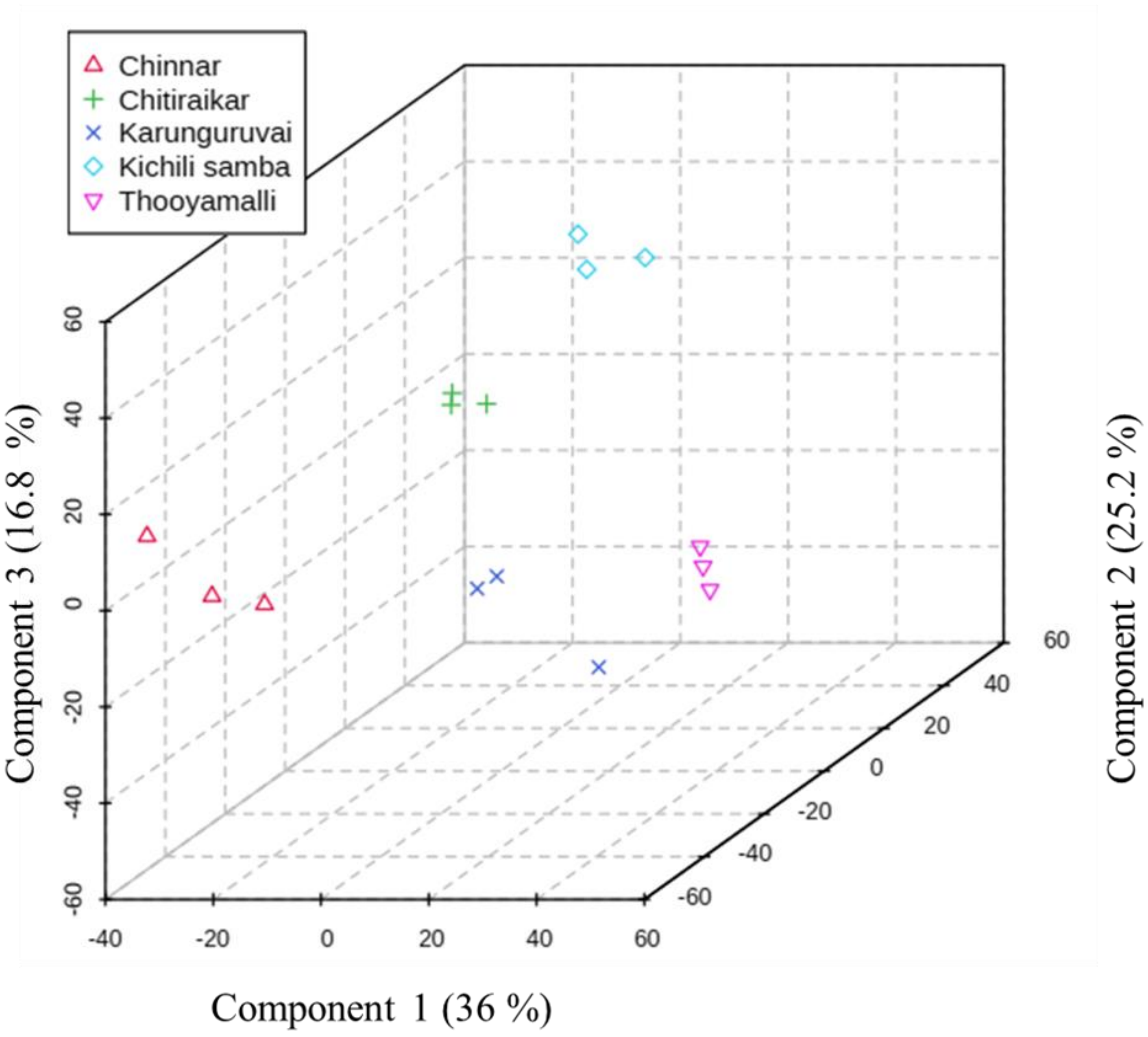

Figure S6. A. 3D of PCA.

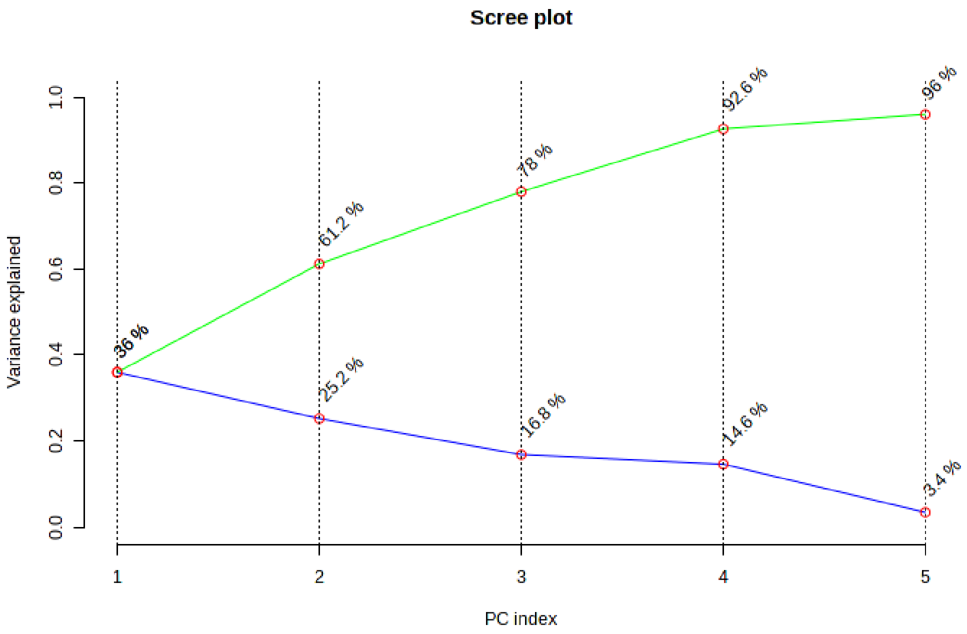

Figure S6. B. Scree plot of PCA.

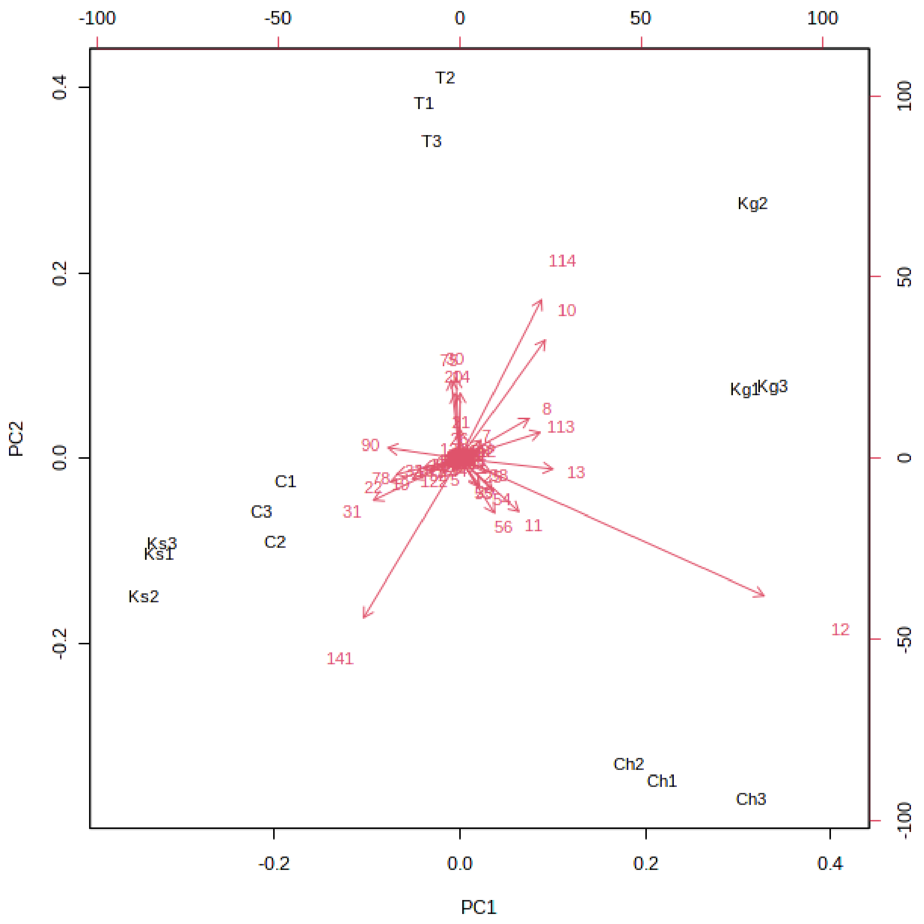

Figure S6.C. PCA biplot between selected PCs.

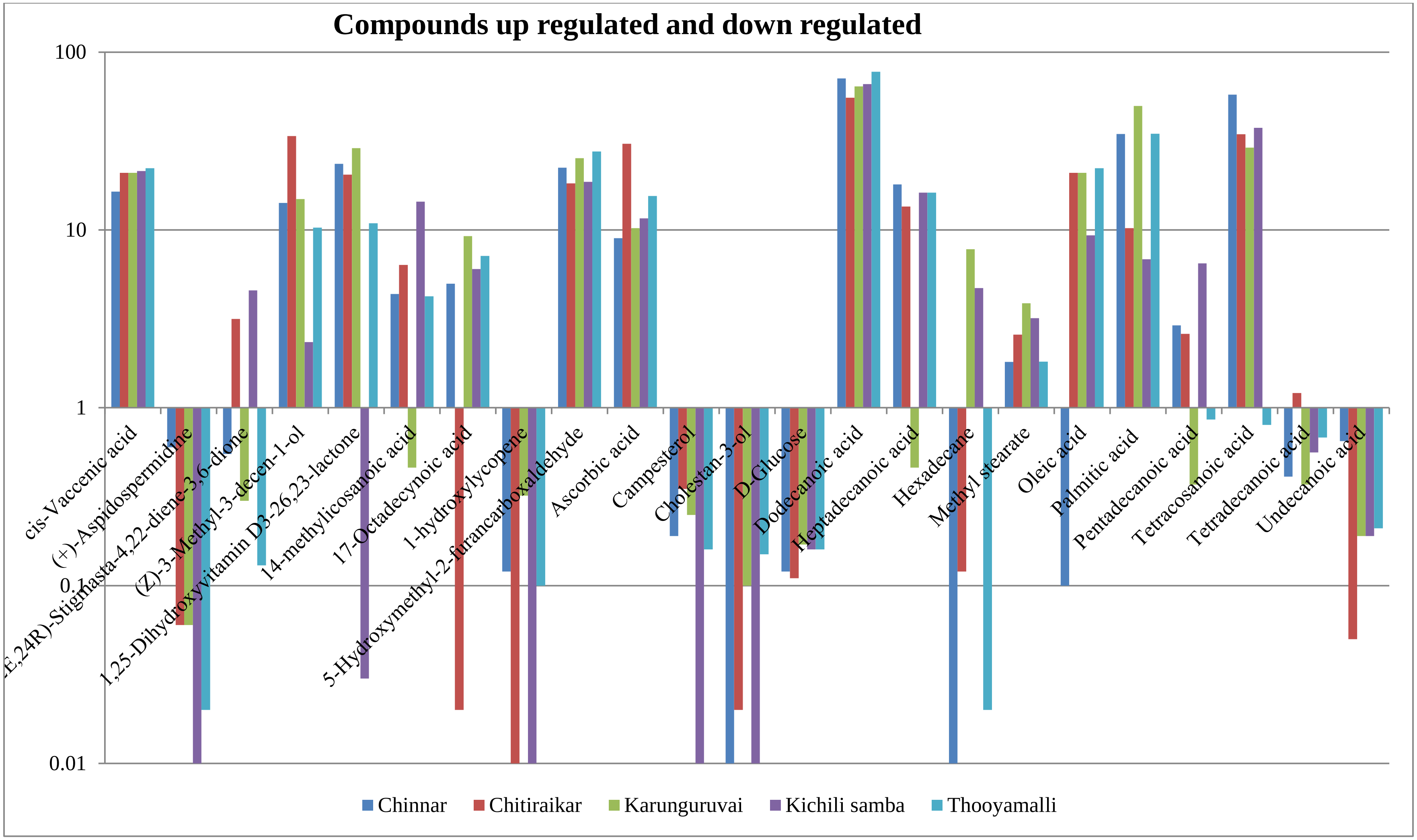

Figures S7. Commonly observed 23 metabolites among the 5 traditional rice varieties along with the up regulated and down regulated status. Values represented are mean of area intensity obtained from three replications.

Table S1. Identified 149 metabolites based on their composition

Other name	Chinnar	Chitiraikar	Karunguruvai	Kichili samba	Thooyamalli	Class
(+)-Aspidospermidine	0.6	0.06	0.06	0.01	0.02	Aspidospermatan-type alkaloids
(22E,24R)-Stigmasta-4,22-diene-3,6-dione	0.55	3.16	0.3	4.57	0.13	Steroids and steroid derivatives
(2E)-undec-2-enoic acid	-	-	0.17	-	-	Fatty Acyls
(2R*,3R*)-1,2,3-Butanetriol	-	7.36	2.1	-	11.68	Organooxygen compounds
(3beta,4alpha,5alpha,9beta)-4,14-Dimethyl-9,19-cycloergost-24-en-3-ol	-	-	26.33	3.86	1.68	Steroids and steroid derivatives
(Acetyloxy)triphenylstannane	-	-	0.37	-	-	Benzene and substituted derivatives
(Z)-2-decenal	-	0.02	29.11	-	24.05	Organooxygen compounds
(Z)-3-Methyl-3-decen-1-ol	14.2	33.71	14.91	2.34	10.3	Fatty Acyls
1,1-Dimethoxynonane	-	73.75	67.03	-	-	Alkaloids and derivatives
1,25-Dihydroxyvitamin D3-26,23-lactone	23.52	20.43	28.84	0.03	10.89	Steroids and steroid derivatives
1,3-Dioxolane	14.02	-	10.65	-	21.01	Dioxolanes
1,4-Cineole	0.1	0.06	-	-	0.19	Prenol lipids
11b-Hydroxyandrost-4-ene-3,17-dione	0.79	0.05	-	-	0.63	Steroids and steroid derivatives
11Z-Eicosenoic acid	8.47	-	0.11	-	-	Fatty Acyls
13-Heptadecyn-1-ol	-	-	0.03	8.58	-	Fatty Acyls
14-methylicosanoic acid	4.37	6.36	0.46	14.43	4.24	Fatty Acyls
16b-Hydroxyestradiol	-	-	-	-	22.48	Steroids and steroid derivatives
17-Octadecynoic acid	4.99	0.02	9.24	6.03	7.15	Fatty Acyls
18-methylnonadecanoic acid	1.08	-	-	19.82	-	Fatty Acyls
1-Acetoxy-2-hydroxy-16-heptadecen-4-one	0.01	-	4.34	-	-	Fatty Acyls

1-Deoxy-D-glucitol	0.03	-	-	-	-	Organooxygen compounds
1-Hexacosene	-	-	-	-	1.03	Unsaturated hydrocarbons
1-Hexadecanethiol	-	8.46	0.3	-	4.86	Thiols
1-Hexadecanol	-	-	0.13	-	4.92	Fatty Acyls
1-hydroxylycopene	0.12	0.01	0.32	0.01	0.1	Prenol lipids
1-Methylhistamine	-	-	0.12	-	-	Organonitrogen compounds
1-Triacontanol	-	6.47	0.27	-	-	Fatty Acyls
2,4-Di-tert-butylphenol	0.18	0.53	0.31	-	23.52	Benzene and substituted derivatives
24-Methylenecycloartan-3-ol	-	4.14	-	29.67	1.68	Steroids and steroid derivatives
2-Furoic acid	-	-	0.1	-	-	Furans
2-Methoxy-4-vinylphenol	0.23	0.18	-	0.12	0.35	Phenols
2-Methylhexacosane	-	-	-	-	0.57	Saturated hydrocarbons
2-Phenylethyl beta-D-glucopyranoside	-	-	0.19	-	-	Organooxygen compounds
2-tert-Butyl-1,4-benzenediol	-	-	0.01	-	-	Benzene and substituted derivatives
3,5-Dimethylpyrazole	-	-	0.07	-	0.07	Azoles
3-Hydroxydodecanoic acid	12.05	25.02	0.03	-	21.84	Hydroxy acids and derivatives
3-Methyltetradecane	-	0.05	-	-	-	Saturated hydrocarbons
3-O-Methyl-d-glucose	-	-	-	-	0.22	Fatty Acyls
3-Palmitoyl-sn-glycerol	1.78	1.75	-	0.36	2.28	Glycerolipids
4-Ethylbenzoic acid	-	-	0.01	-	-	Benzene and substituted derivatives
4-Oxononanal	0.06	-	-	-	-	Organooxygen compounds
4-Phenylpyridine	-	-	-	-	0.06	Pyridines and derivatives
5-Hydroxymethyl-2-furancarboxaldehyde	22.4	18.26	25.36	18.67	27.6	Organooxygen compounds
5Z-Dodecenoic acid	-	-	0.01	-	-	Fatty Acyls
7,7',8,8'-Tetrahydrolycopene	-	-	-	-	0.05	Prenol lipids

9-Undecenal	0.02	-	-	-	-	Organooxygen compounds
Allose	-	-	-	-	0.35	Organooxygen compounds
all-trans-Retinoic acid	0.5	-	-	-	0.63	Prenol lipids
Allyl alcohol	-	-	-	-	0.25	Organooxygen compounds
alpha-Sitosterol	2.13	5.88	-	6.32	2.4	Steroids and steroid derivatives
Ascaridole	0.02	-	-	0.13	-	Dioxanes
Ascorbic acid	8.98	30.52	10.23	11.62	15.5	Dihydrofurans
Azulene	-	-	-	0.38	-	Unsaturated hydrocarbons
Benzothiazole	3.17	15.12	1.23	-	-	Benzothiazoles
Brassicasterol	-	-	-	0.02	-	Steroids and steroid derivatives
Calcitriol	-	-	0.1	-	-	Steroids and steroid derivatives
Campesterol	0.19	0.35	0.25	0.01	0.16	Steroids and steroid derivatives
Campesteryl linoleate	-	1.13	-	-	-	Steroids and steroid derivatives
Capric acid	0.15	-	-	-	-	Fatty Acyls
Cellobiose	-	-	-	-	0.27	Organooxygen compounds
Cholestan-3-ol	0.01	0.02	0.1	0.01	0.15	Steroids and steroid derivatives
Cholic acid	-	0.26	-	-	-	Steroids and steroid derivatives
cis-Sesquisabinene hydrate	0.02	-	-	-	-	Prenol lipids
cis-Vaccenic acid	16.42	20.98	20.93	21.45	22.27	Fatty Acyls
Cycloartenol	-	-	0.38	0.49	-	Steroids and steroid derivatives
Cymbopogonol	-	0.29	-	-	-	Prenol lipids
Dehydroabietic acid	0.79	-	-	-	-	Prenol lipids

Dehydroandrosterone	1.97	-	-	-	-	Steroids and steroid derivatives
Deoxyspergualin	-	-	0.01	-	-	Carboxylic acids and derivatives
Desmosterol	-	-	0.16	-	0.13	Steroids and steroid derivatives
D-Fructose	0.03	0.12	-	-	-	Organooxygen compounds
D-Glucose	0.12	0.11	0.17	0.16	0.16	Organooxygen compounds
Digitoxin	0.03		0.03	0.11	0.13	Steroids and steroid derivatives
Dodecanoic acid	71	55.32	64.23	66.12	77.53	Fatty Acyls
Eicosane	1.04	0.45	-	-	-	Saturated hydrocarbons
Eicosapentaenoic acid	-	-	-	0.04	-	Fatty Acyls
Elaidic acid	0.32	-	-	21.45	2.96	Fatty Acyls
Erucic acid	0.04	0.01	-	-	-	Fatty Acyls
Ethanethioic acid	0.02	-	-	-	-	Carbothioic S-acids
Ethoxyquin	-	-	-	0.05	-	Quinolines and derivatives
Ethyl (4Z)-4,7-octadienoate	-	-	0.32	-	-	Fatty Acyls
Ethyl hexadecanoate	3.32	-	-	-	-	Fatty Acyls
Filbertone	-	-	-	-	0.44	Organooxygen compounds
gamma-Tocopherol	-	0.14	-	-	-	Prenol lipids
Geranylgeranyl-PP	0.01	-	0.12	-	-	Prenol lipids
Glycine	-	-	0.58	-		Carboxylic acids and derivatives
Glycocholic acid	-	-	0.27	-	0.1	Steroids and steroid derivatives
Heneicosanoic acid	4.8	5.13	0.11	-	5.82	Fatty Acyls
Heptadecanoic acid	18.06	13.55	0.46	16.2	16.22	Fatty Acyls
Heptafluorobutyric acid	2.89	-	-	-	-	Alkyl halides
Hexadecane	0.01	0.12	7.8	4.71	0.02	Saturated hydrocarbons

Inosine	-	-	0.56	-	-	Purine nucleosides
Isopentacosane	-	-	0.11	-	-	Saturated hydrocarbons
Isophthalamide	-	-	1.09	-	-	Benzene and substituted derivatives
isosorbide	-	-	-	0.16	-	Furofurans
L-Chlorozotocin	-	-	0.01	-	-	Organooxygen compounds
L-Galactose	-	-	-	-	0.05	Organooxygen compounds
Longispinogenin	-	0.17	-	-	-	Prenol lipids
lupinine	-	0.26	-	-	-	Lupin alkaloids
Lysylvaline	0.01	-	-	-	-	Carboxylic acids and derivatives
Melezitose	0.01	0.02	-	0.02	0.01	Organooxygen compounds
Melibiose	0.01	-	-	-	-	Organooxygen compounds
Methyl 10-undecenoate	-	-	0.34	-	-	Fatty Acyls
Methyl stearate	1.81	2.58	3.87	3.19	1.82	Fatty Acyls
Methyl tetradecanoate	-	-	0.25	0.35	-	Fatty Acyls
Monoethylhexyl phthalic acid	-	-	1.09	-	-	Benzene and substituted derivatives
Myristoleic acid	0.01	-	0.01	-	0.01	Fatty Acyls
N-Dodecane	0.03	-	-	-	-	Saturated hydrocarbons
Nonadecane	1.04	-	5.69	-	-	Saturated hydrocarbons
Nonanal	-	0.04	3.6	0.04	-	Organooxygen compounds
o-Cresol	-	-	0.37	-	-	Phenols
Octadecane	-	0.01	10.19	3.97	-	Saturated hydrocarbons
Oleic acid	0.1	20.98	20.93	9.33	22.27	Fatty Acyls
Palmitic acid	34.6	10.23	49.83	6.84	34.76	Fatty Acyls
Palmitoleic acid	0.06	-	-	-	-	Fatty Acyls
Palmitoyl chloride	-	-	1.08	-	-	Acyl halides
Panaxydol	-	-	-	0.03	-	Fatty Acyls

Paromomycin	-	-	-	-	0.01	Organooxygen compounds
Paullinic acid	3.32	-	0.01	-	-	Fatty Acyls
Pelargonic acid	0.07	-	-	-	0.06	Fatty Acyls
Pentadecane	4.28	3.26	-		4.13	Saturated hydrocarbons
Pentadecanoic acid	2.91	2.6	0.37	6.48	0.86	Fatty Acyls
Petasalbin	-	-	-	-	0.04	Prenol lipids
Phosphonoacetate	-	-	-	0.07	-	Organic phosphonic acids and derivatives
Pregnanediol	0.05	-	-	-	0.13	Steroids and steroid derivatives
Pregnenolone	-	-	-	-	0.06	Steroids and steroid derivatives
Pteleine	-	-	0.1	-	-	Quinolines and derivatives
Rhamnitol	-	-	-	-	0.22	Organooxygen compounds
Ribavirin	-	0.15	-	-	-	Triazole ribonucleosides and ribonucleotides
Sakebiose	-	-	0.14	-	-	Organooxygen compounds
Sinigrin	0.02	-	-	-	0.01	Organooxygen compounds
Smilagenone	-	-	0.1	-	-	Prenol lipids
solasodine 3-O-beta-D-glucopyranoside	-	-	-	0.01	-	Steroids and steroid derivatives
Squalene	2.11	1.39	0.12	1.42	0.28	Prenol lipids
Stearaldehyde	-	-	0.01	-	-	Fatty Acyls
Stigmasterol	-	3.16	-	4.57	2.22	Steroids and steroid derivatives
Stigmasteryl glucoside	-	0.11	-	0.27	0.13	Steroids and steroid derivatives
Sugiol	0.79	-	-	-	-	Prenol lipids
Terephthalic acid	1.71	-	-	-	-	Benzene and substituted derivatives

Testolactone	0.71	-	-	-	-	Naphthopyrans
Tetracosanoic acid	57.6	34.56	29.03	37.46	0.8	Fatty Acyls
Tetradecanoic acid	0.41	1.21	0.37	0.56	0.68	Fatty Acyls
Tridecanoic acid	0.64	-	0.37	-	-	Fatty Acyls
Trigonelline	-	-	-	0.05	-	Alkaloids and derivatives
undec-5-enedioic acid	0.01	-	-	-	-	Fatty Acyls
Undecanoic acid	0.65	0.05	0.19	0.19	0.21	Fatty Acyls
Uric acid	0.02	-	0.02	-	0.08	Imidazopyrimidines
Ursodeoxycholic acid	-	-	-	0.06	-	Steroids and steroid derivatives
Vitamin D3	-	0.02	-	0.14	-	Steroids and steroid derivatives
Xanthotoxol arabinoside	-	0.01	1.17	0.03	-	Coumarins and derivatives
**Total yield of volatile compounds (%)**	**358.59**	**440.16**	**459.78**	**333.01**	**417.93**	

Table S2. Identified 149 metabolites along with retention time for individual variety

Avg. RT	Othername	Chinnar (RT)	Chitiraikar (RT)	Karunguruvai (RT)	Kichili samba (RT)	Thooyamalli (RT)
19.70	(+)-Aspidospermidine	21.53	20.55	19.42	18.4	19.45
28.65	(22E,24R)-Stigmasta-4,22-diene-3,6-dione	30.79	23.19	30.81	23.19	29.82
17.99	(2E)-undec-2-enoic acid			17.99		
7.78	(2R*,3R*)-1,2,3-Butanetriol		8.03	7.96		7.34
29.15	(3beta,4alpha,5alpha,9beta)-4,14-Dimethyl-9,19-cycloergost-24-en-3-ol				29.05	29.25
21.59	(Acetyloxy)triphenylstannane			21.59		
18.29	(Z)-2-decenal		18.55	18.55		18.02
20.18	(Z)-3-Methyl-3-decen-1-ol	19.68	17.59	18.23	21.05	21.74
18.68	1,1-Dimethoxynonane		18.68	18.68		
21.91	1,25-Dihydroxyvitamin D3-26,23-lactone	20.14	20.63	20.55	20.55	26.38
6.17	1,3-Dioxolane	6.29		5.96		6.27
18.87	1,4-Cineole	18.86	18.86			18.87
24.08	11b-Hydroxyandrost-4-ene-3,17-dione	23.31	31.69			24.85
22.04	11Z-Eicosenoic acid	23.18		20.89		
19.73	13-Heptadecyn-1-ol			19.61	19.84	
25.71	14-methylicosanoic acid		25.23	25.62	26.01	25.98
22.27	16b-Hydroxyestradiol					22.27
4.99	17-Octadecynoic acid		4.31	4.89	5.42	4.65
23.61	18-methylnonadecanoic acid	23.26			23.96	
11.34	1-Acetoxy-2-hydroxy-16-heptadecen-4-one	16.03		6.64		
8.02	1-Deoxy-D-glucitol	8.02				
27.03	1-Hexacosene					27.03
21.53	1-Hexadecanethiol		21.38	21.53		21.67
18.39	1-Hexadecanol			18.43		18.34
26.32	1-hydroxylycopene	27.97	27.48	24.68	26.11	26.53
4.08	1-Methylhistamine			4.08		
27.01	1-Triacontanol		26.98	27.03		
20.20	2,4-Di-tert-butylphenol	20.19	20.19	20.19		20.23

29.04	24-Methylenecycloartan-3-ol		29.06		29.03	29.05
4.02	2-Furoic acid			4.02		
19.02	2-Methoxy-4-vinylphenol	19.02	19.02		19.02	19.02
25.35	2-Methylhexacosane					25.35
17.33	2-Phenylethyl beta-D-glucopyranoside			17.33		
29.71	2-tert-Butyl-1,4-benzenediol			29.71		
4.25	3,5-Dimethylpyrazole			4.2		4.29
17.55	3-Hydroxydodecanoic acid	17.44	17.66	17.88		17.23
#DIV/0!	3-Methyltetradecane		19.61			
20.60	3-O-Methyl-d-glucose					20.6
24.20	3-Palmitoyl-sn-glycerol	24.2	24.20		24.19	24.2
4.97	4-Ethylbenzoic acid			4.97		
18.40	4-Oxononanal	18.4				
19.21	4-Phenylpyridine					19.21
18.28	5-Hydroxymethyl-2-furancarboxaldehyde	18.24	18.24	18.24	18.39	18.26
5.96	5Z-Dodecenoic acid			5.96		
19.28	7,7',8,8'-Tetrahydrolycopene					19.28
15.73	9-Undecenal	15.73				
20.19	Allose					20.19
25.05	all-trans-Retinoic acid	24.85				25.24
8.03	Allyl alcohol					8.03
24.77	alpha-Sitosterol	24.76	24.78		24.77	24.77
18.95	Ascaridole	18.95			18.94	
21.78	Ascorbic acid	21.95	21.12	21.93	21.93	21.95
25.87	Azulene		25.89		25.85	
22.17	Benzothiazole	22.03	22.45	22.04		
20.06	Brassicasterol				20.06	
29.04	Calcitriol			29.04		
25.76	Campesterol	22.42	29.33	29.04	22.26	29.33
23.49	Campesteryl linoleate		23.49			
19.34	Capric acid	19.34				

21.35	Cellobiose					21.35
20.40	Cholestan-3-ol	19.76	19.76	20.63	19.55	21.66
21.59	Cholic acid		21.59			
19.89	cis-Sesquisabinene hydrate	19.89				
21.75	cis-Vaccenic acid	22.6	22.58	19.22	22.57	22.61
29.88	Cycloartenol			31.19	28.57	
28.22	Cymbopogonol		28.22			
23.31	Dehydroabietic acid	23.31				
22.90	Dehydroandrosterone	22.9				
6.06	Deoxyspergualin			6.06		
30.13	Desmosterol			30.44		29.82
7.65	D-Fructose	7.65	8.22			
18.06	D-Glucose	17.99	18.21	17.99	18.14	17.99
21.79	Digitoxin	20.8		21.3	23.61	21.43
19.72	Dodecanoic acid	17.44	20.38	20.38		21.34
25.34	Eicosane	25.34	25.34			
17.33	Eicosapentaenoic acid				17.33	
23.09	Elaidic acid	21.26			22.57	25.43
19.50	Erucic acid	19.5	19.20			
8.70	Ethanethioic acid	8.7				
19.89	Ethoxyquin				19.89	
19.02	Ethyl (4Z)-4,7-octadienoate			19.02		
23.18	Ethyl hexadecanoate	23.18				
24.50	Filbertone					24.5
29.69	gamma-Tocopherol		29.69			
26.26	Geranylgeranyl-PP	26.19		26.32		
23.61	Glycine			23.61		
25.45	Glycocholic acid			24.36		26.53
29.26	Heneicosanoic acid	29.54	28.34	30.13		29.04
25.35	Heptadecanoic acid	24.81	25.13	25.62	25.08	26.12
27.02	Heptafluorobutyric acid	27.02				
18.21	Hexadecane	16.03	21.34	21.53	19.24	16.03

16.72	Inosine			16.72		
30.13	Isopentacosane			30.13		
25.87	Isophthalamide			25.87		
17.99	isosorbide				17.99	
16.79	L-Chlorozotocin			16.79		
16.97	L-Galactose					16.97
27.65	Longispinogenin		27.65			
21.59	lupinine		21.59			
16.97	Lysylvaline	16.97				
17.02	Melezitose	16.22	16.23		18.61	16.24
7.53	Melibiose	7.53				
6.54	Methyl 10-undecenoate			6.54		
22.49	Methyl stearate	22.49	22.49	22.49	22.49	22.49
21.07	Methyl tetradecanoate			21.07	21.06	
25.87	Monoethylhexyl phthalic acid			25.87		
21.57	Myristoleic acid	16.22		29.71		18.77
16.73	N-Dodecane	16.73				
24.94	Nonadecane	25.34		24.53		
16.00	Nonanal		16.04	15.97	16.02	
21.59	o-Cresol			21.59		
17.90	Octadecane		16.76	17.56	18.23	
23.90	Oleic acid	26.19	22.58	22.57	24.23	22.61
20.64	Palmitic acid	20.68	21.94	21.21	19.33	21.32
19.22	Palmitoleic acid	19.22				
22.96	Palmitoyl chloride			22.96		
20.75	Panaxydol				20.75	
7.46	Paromomycin					7.46
23.04	Paullinic acid	23.18		22.9		
18.62	Pelargonic acid	18.61				18.62
20.55	Pentadecane	20.55	20.32			20.78
21.43	Pentadecanoic acid	21.81	21.63	21.2	21.3	21.2
17.60	Petasalbin					17.6

19.06	Phosphonoacetate				19.06	
20.75	Pregnanediol	20.06				21.43
28.79	Pregnenolone					28.79
20.75	Pteleine			20.75		
19.96	Rhamnitol					19.96
18.00	Ribavirin		18.00			
29.83	Sakebiose			29.83		
12.48	Sinigrin	17.55				7.4
20.69	Smilagenone			20.69		
19.20	solasodine 3-O-beta-D-glucopyranoside				19.2	
26.62	Squalene	26.62	26.62	26.62	26.62	26.62
26.32	Stearaldehyde			26.32		
23.19	Stigmasterol		23.19		23.19	23.18
29.82	Stigmasteryl glucoside		29.82		29.82	29.82
23.31	Sugiol	23.31				
25.86	Terephthalic acid	25.86				
24.58	Testolactone	24.58				
25.33	Tetracosanoic acid	25.61	24.69	25.62	25.61	25.12
20.72	Tetradecanoic acid	20.32	20.01	21.2	21.19	20.89
20.79	Tridecanoic acid	20.38		21.2		
16.90	Trigonelline				16.9	
16.22	undec-5-enedioic acid	16.22				
18.77	Undecanoic acid	17.23	18.62	19.33	20.2	18.3
19.80	Uric acid	19.8		19.8		19.81
30.18	Ursodeoxycholic acid				30.18	
20.63	Vitamin D3		29.50		20.63	
26.01	Xanthotoxol arabinoside		29.92	22.09	29.92	

Table S3. Venn daigram results of metabolites commonly present among all the 5 varieties and specific to each rice varieties

Names	Total	Metabolites
Chinnar, Chitiraikar Karunguruvai, Kichili samba, Thooyamalli	23	cis-Vaccenic acid, 5-Hydroxymethyl-2-furancarboxaldehyde, Squalene, D-Glucose, Palmitic acid, "(22E,24R)-Stigmasta-4,22-diene-3,6-dione", Undecanoic acid, 1-hydroxylycopene, Ascorbic acid, Cholestan-3-ol, (Z)-3-Methyl-3-decen-1-ol, Tetracosanoic acid, 17-Octadecynoic acid, "1,25-Dihydroxyvitamin, D3-26,23-lactone", Dodecanoic acid, Pentadecanoic acid, Heptadecanoic acid, 14-methylicosanoic acid, Hexadecane, (+)-Aspidospermidine, Tetradecanoic acid, Methyl stearate ,Campesterol
Chinnar, Chitiraikar, Karunguruvai, Kichili samba	1	Oleic acid
Chinnar, Chitiraikar, Kichili samba, Thooyamalli	4	alpha-Sitosterol, 2-Methoxy-4-vinylphenol, Melezitose, 3-Palmitoyl-sn-glycerol
Chinnar, Karunguruvai, Kichili samba, Thooyamalli	1	Digitoxin
Chinnar, Chitiraikar, Karunguruvai	1	"2,4-Di-tert-butylphenol"
Chinnar, Chitiraikar, Thooyamalli	2	"11b-Hydroxyandrost-4-ene-3,17-dione", "1,4-Cineole "
Chinnar,	3	"1,3-Dioxolane", Uric acid, Myristoleic acid

Karunguruvai, Thooyamalli		
Chinnar, Kichili samba, Thooyamalli	1	Elaidic acid
Chitiraikar, Karunguruvai, Kichili samba	2	Nonanal, Xanthotoxol arabinoside
Chitiraikar, Kichili samba, Thooyamalli	3	Stigmasterol, Stigmasteryl glucoside,"(3beta,4alpha,5alpha,9beta)-4,14-Dimethyl-9,19-cycloergost-24-en-3-ol"
Chinnar, Chitiraikar	3	Erucic acid, Eicosane, D-Fructose
Chinnar, Karunguruvai,	6	Paullinic acid, "2,6-Di-tert-butyl-4-ethylphenol", Tridecanoic acid, Benzothiazole, 3-Hydroxydodecanoic acid, 1-Acetoxy-2-hydroxy-16-heptadecen-4-one
Chinnar, Kichili samba	1	Ascaridole
Chinnar, Thooyamalli	4	Sinigrin, Pelargonic acid, Pregnanediol, all-trans-Retinoic acid
Chitiraikar, Karunguruvai	2	"1,1-Dimethoxynonane" (Z)-2-decenal
Chitiraikar, Kichili samba	1	Vitamin D3
Chitiraikar, Thooyamalli	1	24-Methylenecycloartan-3-ol
Karunguruvai, Kichili	2	Cycloartenol, Methyl tetradecanoate

samba		
Karunguruvai, Thooyamalli	3	Glycocholic acid, "3,5-Dimethylpyrazole", Desmosterol
Chinnar	22	Heptafluorobutyric acid, Palmitoleic acid, 4-Oxononanal, Nonadecane, Melibiose, Ethanethioic acid, Geranyl-PP Ethyl, hexadecanoate, Capric acid, Pentadecane, 1-Deoxy-D-glucitol, Terephthalic acid, N-Dodecane, Lysylvaline, Testolactone, undec-5-enedioic acid, Sugiol, Dehydroabietic acid, Dehydroandrosterone, 18-methylnonadecanoic acid, 9-Undecenal cis-Sesquisabinene hydrate
Chitiraikar	9	Campesteryl linoleate, Cymbopogonol, lupinine, Longispinogenin, 3-Methyltetradecane, gamma-Tocopherol, Cholic acid, Ribavirin, Octadecane
Karunguruvai	33	Monoethylhexyl phthalic acid, Pteleine, o-Cresol, 1-Triacontanol, 1-Methylhistamine, Smilagenone, Methyl 10-undecenoate, 4-Ethylbenzoic acid, Isophthalamide, Calcitriol, "Ethyl (4Z)-4,7-octadienoate", Palmitoyl chloride, 1-Hexadecanethiol, L-Chlorozotocin, Heneicosanoic acid, 13-Heptadecyn-1-ol, Glycine, Deoxyspergualin, "2-tert-Butyl-1,4-benzenediol", (Acetyloxy)triphenylstannane, Inosine, 2-Furoic acid, Geranylgeranyl-PP, (2E)-undec-2-enoic acid, "(2R*,3R*)-1,2,3-Butanetriol", Sakebiose, Dibutyl phthalate, 2-Phenylethyl, beta-D-glucopyranoside, 5Z-Dodecenoic acid, Stearaldehyde, Isopentacosane, 1-Hexadecanol, 11Z-Eicosenoic acid
Kichili samba	13	Panaxydol, 4-Phenylpyridine, "7,7',8,8'-Tetrahydrolycopene", Phosphonoacetate, Brassicasterol, Eicosapentaenoic acid, Trigonelline, Ursodeoxycholic acid, Azulene, isosorbide, solasodine, 3-O-beta-D-glucopyranoside, Ethoxyquin, Filbertone
Thooyamalli	13	Rhamnitol, L-Galactose, Petasalbin, Oleic Acid, 1-Hexacosene, Cellobiose, Pregnenolone, Allose Allyl alcohol, 16b-Hydroxyestradiol, 2-Methylhexacosane, Paromomycin, 3-O-Methyl-d-glucose

Table S4. ANOVA for significant metabolites

Metabolites	f.value	p.value	-log10(p)	FDR
decenal	447.39	3.16E-11	10.5	4.71E-09
Eicosenoic acid	311.86	1.90E-10	9.7222	1.06E-08
Heptadecynol	304.24	2.14E-10	9.669	1.06E-08
Methylenecycloartanol	255.53	5.08E-10	9.294	1.89E-08
Ditert butylphenol	227.56	9.01E-10	9.0453	2.68E-08
Hydroxyestradiol	182.14	2.70E-09	8.5687	6.70E-08
Terephthalic acid	166.23	4.23E-09	8.3735	8.78E-08
Tridecanoic acid	162.6	4.72E-09	8.3265	8.78E-08
Dimethylcycloergostenol	155.52	5.87E-09	8.2315	9.72E-08
Palmitic acid	129.87	1.42E-08	7.848	2.11E-07
Azulene	123.19	1.84E-08	7.7358	2.49E-07
Dimethoxynonane	108.75	3.37E-08	7.4719	4.19E-07
methylnonadecanoic acid	91.563	7.78E-08	7.109	8.92E-07
Hydroxydodecanoic acid	83.276	1.23E-07	6.9096	1.22E-06
Nonadecane	83.148	1.24E-07	6.9064	1.22E-06
Oleic acid	82.223	1.31E-07	6.883	1.22E-06
Dioxolane	80.392	1.46E-07	6.8358	1.28E-06
Hexadecane	67.556	3.37E-07	6.4725	2.79E-06
Methylhexacosane	64.474	4.21E-07	6.3754	3.23E-06
Elaidic acid	64.069	4.34E-07	6.3623	3.23E-06
Paullinic acid	60.719	5.61E-07	6.2509	3.76E-06
Heptafluorobutyric acid	60.592	5.67E-07	6.2466	3.76E-06
methylicosanoic acid	60.279	5.81E-07	6.2359	3.76E-06
Triacontanol	56.572	7.86E-07	6.1046	4.88E-06
Pentadecanoic acid	56	8.25E-07	6.0837	4.92E-06
lupinine	49.367	1.50E-06	5.8244	8.59E-06

Ethyl hexadecanoate	46.92	1.90E-06	5.7204	1.05E-05
Acetyloxy triphenylstannane	45.761	2.14E-06	5.6693	1.14E-05
Dehydroandrosterone	37.764	5.25E-06	5.2798	2.70E-05
Pentadecane	36.097	6.47E-06	5.189	3.21E-05
Acetoxy hydroxy heptadecenone	32.786	1.01E-05	4.9966	4.84E-05
Stigmasta diene 6 dione	30.954	1.31E-05	4.8823	6.11E-05
Methyl decenol	27.309	2.32E-05	4.6353	1.05E-04
Octadecynoic acid	27.108	2.39E-05	4.6209	1.05E-04
Hexadecanethiol	26.203	2.79E-05	4.5545	1.18E-04
Sugiol	26.069	2.85E-05	4.5445	1.18E-04
Isophthalamide	24.862	3.53E-05	4.4523	1.41E-04
Dihydroxyvitamin lactone	24.751	3.60E-05	4.4436	1.41E-04
Palmitoyl chloride	22.903	5.08E-05	4.2937	1.94E-04
Heneicosanoic acid	22.504	5.50E-05	4.26	1.98E-04
Nonanal	22.501	5.50E-05	4.2597	1.98E-04
Octadecane	22.417	5.59E-05	4.2525	1.98E-04
all trans Retinoic acid	19.978	9.26E-05	4.0332	3.21E-04
Methyl undecenoate	19.864	9.50E-05	4.0224	3.22E-04
Xanthotoxol arabinoside	19.304	1.08E-04	3.9684	3.56E-04
Ascorbic acid	18.73	1.23E-04	3.9118	3.97E-04
Longispinogenin	18.253	1.37E-04	3.8635	4.31E-04
Tetracosanoic acid	18.197	1.39E-04	3.8577	4.31E-04
Filbertone	17.463	1.66E-04	3.7811	5.03E-04
Benzothiazole	15.952	2.43E-04	3.6144	7.24E-04
Sakebiose	15.865	0.00025	3.6044	0.00073
Allose	13.421	0.0005	3.3029	0.00143
Monoethylhexyl phthalic acid	13.18	0.00054	3.2708	0.00151
Cresol	12.738	0.00062	3.2107	0.0017

Stigmasterol	12.233	0.00072	3.1398	0.00196
Heptadecanoic acid	11.61	0.00089	3.0492	0.00238
Cymbopogonol	11.035	0.00109	2.9621	0.00285
Campesteryl linoleate	10.155	0.00151	2.8215	0.00387
Eicosane	10.104	0.00154	2.8132	0.00388
alpha Sitosterol	9.3792	0.00204	2.6898	0.00507
Ethyl octadienoate	8.9086	0.00248	2.6059	0.00605
Capric acid	8.2514	0.00329	2.483	0.0079
Allyl alcohol	8.104	0.00351	2.4545	0.00831
Tetradecanoic acid	7.9626	0.00374	2.4268	0.00871
Pregnanediol	7.5769	0.00447	2.3494	0.01025
Cellobiose	7.493	0.00465	2.3322	0.01051
Geranylgeranyl PP	7.4282	0.0048	2.3188	0.01067
Methyl d glucose	7.3691	0.00494	2.3065	0.01082
undecenoic acid	7.1195	0.00557	2.2539	0.01203
Cycloartenol	7.0219	0.00585	2.233	0.01245
Ribavirin	6.9411	0.00609	2.2155	0.01278
Hydroxyandrostene	6.4843	0.00769	2.1141	0.01591
Cholic acid	6.451	0.00782	2.1066	0.01597
Squalene	6.3066	0.00844	2.0734	0.017
Testolactone	6.2677	0.00862	2.0644	0.01713
Palmitoylsn glycerol	6.157	0.00915	2.0385	0.01794
Ethoxyquin	5.92	0.01042	1.9822	0.0199
Smilagenone	5.92	0.01042	1.9822	0.0199
Hexadecanol	5.8066	0.0111	1.9547	0.02094
Pteleine	5.7442	0.0115	1.9394	0.02141
isosorbide	5.7083	0.01173	1.9306	0.02159
Dehydroabietic acid	5.3979	0.01404	1.8527	0.02533

Methylhistamine	5.3894	0.01411	1.8505	0.02533
Phosphonoacetate	5.2272	0.01553	1.8087	0.02756
Rhamnitol	5.1855	0.01593	1.7978	0.02792
cis Vaccenic acid	5.0867	0.01691	1.7718	0.02912
Phenylethyl beta D glucopyranoside	5.0777	0.017	1.7695	0.02912
Eicosapentaenoic acid	4.956	0.01832	1.737	0.03103
gamma Tocopherol	4.8375	0.01973	1.7049	0.03303
Pelargonic acid	4.7775	0.02049	1.6885	0.03392
Phenylpyridine	4.6132	0.02275	1.643	0.03725
Methyl stearate	4.5632	0.0235	1.6289	0.03806
Vitamin D3	4.5144	0.02426	1.6152	0.03874
Oxononanal	4.5028	0.02444	1.6119	0.03874
Dodecenoic acid	4.32	0.02758	1.5594	0.04326

Table S5. Loadings of the variables in the all principal components.

	**PC1**	**PC2**	**PC3**	**PC4**	**PC5**
**Variation %**	**36**	**25.2**	**16.8**	**14.6**	**3.4**
Deoxy D glucitol	-0.0002	-0.0001	0.0008	-0.0004	-0.0015
cis Vaccenic acid	0.0427	0.0101	**-0.1321**	0.0326	0.0940
undecenedioic acid	-0.0005	-0.0002	0.0019	-0.0009	-0.0048
Aspidospermidine	0.0000	0.0055	0.0075	0.0024	-0.0010
Stigmasta diene 6 dione	-0.0103	-0.0506	-0.0392	0.0086	-0.0221
undecenoic acid	0.0027	0.0019	0.0030	0.0043	-0.0044
Butanetriol	0.0516	0.0513	-0.0693	-0.0818	0.0043
Dimethylcycloergostenol	**0.1664**	**0.1145**	0.0848	**0.3710**	-0.0675
Acetyloxy triphenylstannane	0.0027	0.0016	0.0017	0.0043	-0.0007
decenal	**0.2054**	**0.3393**	0.0093	**0.1542**	-0.0259
Methyl decenol	**0.1416**	**-0.1529**	0.0380	-0.2363	0.0106
Dimethoxynonane	**0.7312**	**-0.3939**	-0.0558	**0.1691**	-0.0918
Dihydroxyvitamin lactone	**0.2238**	**-0.0303**	**0.2569**	**-0.1010**	**0.1062**
Dioxolane	0.0013	**0.1874**	0.0917	**-0.1037**	0.0560
Cineole	0.0002	0.0002	0.0007	-0.0066	-0.0066
Hydroxyandrostene	-0.0013	0.0035	0.0010	-0.0081	0.0125
Eicosenoic acid	-0.0404	-0.0136	**0.1280**	-0.0468	0.0264
Heptadecynol	-0.0680	-0.0283	-0.0658	0.0953	-0.0440
methylicosanoic acid	**-0.1131**	-0.0584	**-0.1026**	0.0619	-0.0934
Hydroxyestradiol	-0.0121	**0.1863**	**-0.1152**	**-0.1631**	0.0051
Octadecynoic acid	0.0015	0.0821	0.0366	0.0929	-0.0364
methylnonadecanoic acid	**-0.1663**	-0.0677	**-0.1514**	**0.2167**	-0.0905
Acetoxy hydroxy heptadecenone	0.0451	0.0237	0.0301	0.0714	-0.0066
Hexacosene	-0.0008	0.0105	-0.0070	-0.0099	-0.0005
Hexadecanethiol	0.0623	-0.0409	-0.0992	**-0.1346**	-0.0172

Hexadecanol	0.0001	0.0433	-0.0221	-0.0296	0.0042
hydroxylycopene	-0.0002	0.0054	0.0048	-0.0022	-0.0065
Methylhistamine	0.0020	0.0010	0.0014	0.0033	-0.0001
Triacontanol	0.0472	-0.0765	-0.0427	-0.0572	-0.0111
Ditert butylphenol	-0.0092	**0.2300**	**-0.1404**	**-0.1998**	0.0108
Methylenecycloartanol	**-0.2072**	**-0.1206**	-0.2671	**0.2513**	**-0.1528**
Furoic acid	0.0023	0.0010	0.0015	0.0036	0.0001
Methoxy vinylphenol	-0.0881	-0.0280	**0.3187**	**-0.1505**	**-0.8916**
Methylhexacosane	-0.0003	0.0047	-0.0029	-0.0041	0.0001
Phenylethyl beta D glucopyranoside	0.0024	0.0010	0.0017	0.0038	0.0001
tertbutyl benzenediol	0.0018	0.0006	0.0013	0.0029	0.0004
Dimethylpyrazole	0.0023	0.0037	-0.0001	0.0015	-0.0004
Hydroxydodecanoic acid	0.0742	-0.0390	**-0.1004**	**-0.5128**	-0.0180
Methyltetradecane	0.0025	-0.0036	-0.0024	-0.0026	-0.0021
Methyl d glucose	-0.0002	0.0035	-0.0022	-0.0032	0.0000
Palmitoylsn glycerol	0.0019	0.0027	-0.0147	-0.0621	-0.0013
Ethylbenzoic acid	0.0022	0.0010	0.0014	0.0035	-0.0001
Oxononanal	-0.0029	-0.0013	0.0093	-0.0038	-0.0011
Phenylpyridine	-0.0002	0.0032	-0.0019	-0.0029	0.0003
Hydroxymethyl furancarboxaldehyde	**0.0298**	-0.0062	**0.1172**	-0.0799	**-0.1006**
Dodecenoic acid	0.0004	0.0002	0.0002	0.0006	0.0000
Tetrahydrolycopene	-0.0003	0.0034	-0.0025	-0.0036	-0.0003
Undecenal	-0.0008	-0.0003	0.0031	-0.0014	-0.0072
Allose	-0.0003	0.0059	-0.0037	-0.0054	0.0002
all trans Retinoic acid	-0.0022	0.0069	0.0011	-0.0085	0.0002
Allyl alcohol	-0.0004	0.0062	-0.0039	-0.0057	0.0003
alpha Sitosterol	-0.0421	-0.0399	-0.0812	-0.0039	-0.0141
Ascaridole	-0.0021	0.0043	0.0033	-0.0074	-0.0096

Ascorbic acid	0.0798	-0.0927	**-0.2155**	**-0.1692**	-0.0878
Azulene	0.0456	-0.0818	-0.0473	-0.0602	-0.0113
Benzothiazole	0.0834	**-0.1568**	-0.0281	**-0.1285**	-0.0259
Brassicasterol	-0.0012	-0.0005	-0.0015	0.0018	-0.0013
Calcitriol	0.0016	0.0013	0.0008	0.0026	-0.0011
Campesterol	0.0017	0.0010	-0.0019	-0.0070	-0.0087
Campesteryl linoleate	0.0112	-0.0183	-0.0108	-0.0142	-0.0043
Capric acid	-0.0012	-0.0004	0.0038	-0.0016	-0.0031
Cellobiose	-0.0004	0.0059	-0.0038	-0.0055	0.0001
Cholestanol	-0.0018	0.0034	-0.0010	-0.0055	-0.0122
Cholic acid	0.0028	-0.0051	-0.0029	-0.0043	-0.0002
cis Sesquisabinene hydrate	-0.0010	-0.0004	0.0032	-0.0014	-0.0033
Cycloartenol	-0.0046	-0.0020	-0.0048	0.0127	-0.0042
Cymbopogonol	0.0032	-0.0054	-0.0031	-0.0043	-0.0009
Dehydroabietic acid	-0.0026	-0.0010	0.0071	-0.0023	0.0178
Dehydroandrosterone	-0.0090	-0.0031	0.0281	-0.0111	-0.0062
Deoxyspergualin	0.0023	0.0007	0.0017	0.0037	0.0006
Desmosterol	0.0037	0.0078	-0.0008	0.0014	0.0004
D Fructose	0.0013	-0.0050	0.0027	-0.0060	-0.0147
D Glucose	0.0008	-0.0012	-0.0009	0.0011	0.0002
Digitoxin	-0.0069	0.0016	-0.0054	0.0185	-0.0182
Dodecanoic acid	-0.0207	**0.2239**	0.0147	0.0481	0.0233
Eicosane	-0.0066	-0.0076	0.0267	-0.0153	-0.0165
Eicosapentaenoic acid	-0.0015	-0.0007	-0.0015	0.0020	-0.0010
Elaidic acid	**-0.1512**	-0.0457	**-0.1373**	**0.1733**	-0.0905
Erucic acid	-0.0002	-0.0006	0.0015	-0.0011	-0.0034
Ethanethioic acid	-0.0010	-0.0003	0.0036	-0.0016	-0.0083
Ethoxyquin	-0.0004	-0.0001	-0.0004	0.0005	-0.0003

Ethyl octadienoate	0.0035	0.0016	0.0023	0.0055	-0.0001
Ethyl hexadecanoate	-0.0160	-0.0059	0.0484	-0.0186	0.0215
Filbertone	-0.0003	0.0053	-0.0034	-0.0048	0.0001
gamma Tocopherol	0.0027	-0.0043	-0.0026	-0.0033	-0.0013
Geranylgeranyl PP	0.0022	0.0015	0.0021	0.0035	-0.0013
Glycine	0.0009	0.0006	0.0004	0.0066	-0.0006
Glycocholic acid	0.0018	0.0035	-0.0001	0.0008	0.0007
Heneicosanoic acid	0.0011	0.0063	0.0067	-0.0978	0.0770
Heptadecanoic acid	**-0.1720**	0.0299	0.0122	**-0.1854**	0.0786
Heptafluorobutyric acid	-0.0128	-0.0044	0.0400	-0.0156	-0.0029
Hexadecane	0.0120	0.0049	-0.0025	**0.1192**	-0.0358
Isopentacosane	0.0022	0.0010	0.0015	0.0036	0.0001
Isophthalamide	0.0097	0.0051	0.0063	0.0154	-0.0012
isosorbide	-0.0026	-0.0012	-0.0024	0.0033	-0.0015
Chlorozotocin	0.0015	0.0005	0.0011	0.0023	0.0003
Galactose	-0.0001	0.0011	-0.0008	-0.0011	0.0000
Longispinogenin	0.0012	-0.0020	-0.0012	-0.0015	-0.0004
lupinine	0.0012	-0.0021	-0.0012	-0.0017	0.0000
Lysylvaline	-0.0011	-0.0003	0.0040	-0.0018	-0.0105
Melezitose	-0.0003	-0.0027	0.0003	-0.0046	0.0014
Melibiose	-0.0008	-0.0002	0.0028	-0.0013	-0.0072
Methyl undecenoate	0.0025	0.0011	0.0017	0.0039	0.0000
Methyl stearate	0.0220	-0.0107	-0.0054	0.0055	0.0078
Methyl tetradecanoate	-0.0017	-0.0008	-0.0017	0.0075	-0.0015
Monoethylhexyl phthalic acid	0.0086	0.0039	0.0060	0.0137	0.0001
Myristoleic acid	0.0011	0.0009	0.0012	0.0015	0.0006
Dodecane	-0.0022	-0.0010	0.0066	-0.0026	0.0044
Nonadecane	0.0295	0.0153	0.0413	0.0479	-0.0136

Nonanal	0.0219	0.0111	0.0108	0.0392	-0.0090
Cresol	0.0037	0.0018	0.0025	0.0058	-0.0002
Octadecane	0.0421	0.0181	0.0157	**0.1792**	-0.0284
Oleic acid	**0.1930**	0.0741	**-0.2184**	-0.1002	-0.0549
Palmitic acid	**0.1966**	**0.4543**	0.3992	0.0501	0.0250
Palmitoleic acid	-0.0009	-0.0003	0.0032	-0.0014	-0.0067
Palmitoyl chloride	0.0096	0.0059	0.0058	0.0152	-0.0029
Panaxydol	-0.0020	-0.0008	-0.0021	0.0028	-0.0018
Paromomycin	-0.0001	0.0028	-0.0016	-0.0023	0.0005
Paullinic acid	-0.0177	-0.0065	0.0591	-0.0200	0.0235
Pelargonic acid	-0.0006	0.0004	0.0011	-0.0011	0.0018
Pentadecane	-0.0127	0.0199	0.0245	-0.0820	0.0320
Pentadecanoic acid	-0.0506	-0.0526	**-0.0362**	0.0374	-0.0212
Petasalbin	-0.0001	0.0021	-0.0015	-0.0022	-0.0001
Phosphonoacetate	-0.0005	-0.0002	-0.0005	0.0007	-0.0004
Pregnanediol	-0.0009	0.0020	0.0009	-0.0031	-0.0003
Pregnenolone	-0.0002	0.0022	-0.0016	-0.0023	-0.0002
Pteleine	0.0010	0.0004	0.0007	0.0015	0.0001
Rhamnitol	-0.0002	0.0031	-0.0021	-0.0030	-0.0001
Ribavirin	0.0016	-0.0029	-0.0016	-0.0024	-0.0001
Sakebiose	0.0015	0.0009	0.0009	0.0024	-0.0005
Sinigrin	-0.0010	0.0006	0.0029	-0.0025	-0.0081
Smilagenone	0.0018	0.0009	0.0012	0.0029	-0.0002
solasodine beta D glucopyranoside	-0.0004	-0.0002	-0.0005	0.0006	-0.0004
Squalene	-0.0122	-0.0295	0.0005	-0.0113	0.0002
Stearaldehyde	0.0018	0.0006	0.0013	0.0029	0.0004
Stigmasterol	-0.0262	-0.0128	-0.0815	0.0042	-0.0349
Stigmasteryl glucoside	-0.0032	-0.0011	-0.0097	-0.0003	-0.0034

Sugiol	-0.0030	-0.0011	0.0087	-0.0031	0.0105
Terephthalic acid	-0.0070	-0.0025	0.0209	-0.0079	0.0104
Testolactone	-0.0020	-0.0007	0.0055	-0.0018	0.0131
Tetracosanoic acid	**-0.2311**	**-0.4568**	**0.5364**	0.0321	**0.2648**
Tetradecanoic acid	0.0071	-0.0099	-0.0082	-0.0092	-0.0052
Tridecanoic acid	-0.0004	0.0002	0.0083	0.0005	0.0050
Trigonelline	-0.0019	-0.0007	-0.0022	0.0028	-0.0020
Undecanoic acid	-0.0011	0.0025	0.0033	-0.0012	0.0016
Uric acid	-0.0002	0.0055	-0.0005	-0.0048	-0.0044
Ursodeoxycholic acid	-0.0013	-0.0005	-0.0015	0.0019	-0.0013
Vitamin D3	0.0013	-0.0038	0.0007	-0.0038	-0.0037
Xanthotoxol arabinoside	0.0131	0.0067	0.0075	0.0244	-0.0035

***Bolded loadings are highly weighted variables**

Table S6. Pathways identified using METABOANALYST in 5 rice varieties

Pathway	Total	Expected	Hits	Raw p	-log10(p)	Holm adjust	FDR	Impact
Steroid biosynthesis	42	0.59613	5	0.000215	3.6682	0.018035	0.02	0.03
Fatty acid biosynthesis	47	0.6671	4	0.003638	2.4391	0.30195	0.15	0.01
Biosynthesis of unsaturated fatty acids	36	0.51097	3	0.013088	1.8831	1	0.37	0
Primary bile acid biosynthesis	46	0.6529	3	0.025379	1.5955	1	0.53	0.02
Histidine metabolism	16	0.2271	1	0.20534	0.68752	1	1	0.09
Retinol metabolism	17	0.24129	1	0.21674	0.66406	1	1	0.22
Terpenoid backbone biosynthesis	18	0.25548	1	0.22798	0.6421	1	1	0
Purine metabolism	65	0.92258	2	0.23479	0.62932	1	1	0
Glycolysis / Gluconeogenesis	26	0.36903	1	0.31252	0.50512	1	1	0
Glutathione metabolism	28	0.39742	1	0.33224	0.47855	1	1	0.09
Porphyrin and chlorophyll metabolism	30	0.42581	1	0.35141	0.45419	1	1	0
Glyoxylate and dicarboxylate metabolism	32	0.45419	1	0.37005	0.43174	1	1	0.11
Glycine, serine and threonine metabolism	33	0.46839	1	0.37918	0.42115	1	1	0.25
Amino sugar and nucleotide sugar metabolism	37	0.52516	1	0.41445	0.38252	1	1	0
Fatty acid elongation	39	0.55355	1	0.43136	0.36516	1	1	0
Fatty acid degradation	39	0.55355	1	0.43136	0.36516	1	1	0
Aminoacyl-tRNA biosynthesis	48	0.68129	1	0.50186	0.29942	1	1	0
Steroid hormone biosynthesis	85	1.2065	1	0.71336	0.14669	1	1	0.03

Table S7. VIP scores for metabolites analyzed through PLS-DA

Metabolites	Comp. 1	Comp. 2	Comp. 3
etracosanoic acid	7.6528	7.465	7.4492
Dimethoxynonane	4.0972	4.4099	4.4029
Dihydroxyvitamin lactone	3.2548	3.1936	3.21
Ditert butylphenol	2.9594	2.8829	2.8719
decenal	2.8687	2.7935	2.7839
Hydroxyestradiol	2.4381	2.3747	2.3661
Methyl decenol	2.3614	2.3076	2.3019
Methoxy vinylphenol	2.3488	2.3027	2.3359
Oleic acid	2.104	2.1739	2.1664
Methylenecycloartanol	2.0553	2.0774	2.0921
Dodecanoic acid	1.8707	1.8715	1.8688
Benzothiazole	1.5828	1.5639	1.5603
Elaidic acid	1.4693	1.4662	1.4892
methylnonadecanoic acid	1.3655	1.3542	1.3625
Eicosenoic acid	1.2148	1.2319	1.2276
Hydroxymethyl furancarboxaldehyde	1.1532	1.1339	1.1294
cis Vaccenic acid	1.0532	1.0518	1.0505
Butanetriol	0.76257	0.74625	0.74599
Dioxolane	0.7278	0.84702	0.84555
methylicosanoic acid	0.70856	0.71184	0.72476
Ascorbic acid	0.62939	0.8943	0.89403
Stigmasterol	0.62405	0.6228	0.62149
Heptadecynol	0.62025	0.61968	0.62561
Dimethylcycloergostenol	0.56929	0.56375	0.5784
Octadecynoic acid	0.56466	0.5665	0.56482
Paullinic acid	0.55915	0.56554	0.56346
Azulene	0.5535	0.57607	0.57543
Triacontanol	0.54279	0.56205	0.56173

Hydroxydodecanoic acid	0.53321	0.5229	0.53121
Hexadecanol	0.52512	0.51178	0.50976
Ethyl hexadecanoate	0.46703	0.47291	0.47118
alpha Sitosterol	0.42098	0.44129	0.44576
Palmitic acid	0.41748	1.0998	1.1313
Heptafluorobutyric acid	0.37291	0.37665	0.37518
Octadecane	0.33919	0.38117	0.38676
Hexadecane	0.29195	0.31146	0.31486
Eicosane	0.28889	0.28556	0.28451
Squalene	0.28211	0.27481	0.27386
Heptadecanoic acid	0.26352	0.43344	0.45846
Dehydroandrosterone	0.25972	0.26194	0.26091
Heneicosanoic acid	0.20981	0.23122	0.23549
Terephthalic acid	0.20356	0.20674	0.20606
Nonadecane	0.15247	0.14848	0.14798
Pentadecane	0.14867	0.2035	0.20343
Hexacosene	0.14346	0.1397	0.13929
Campesteryl linoleate	0.12798	0.13235	0.13244
Digitoxin	0.095611	0.093105	0.092756
Methyl stearate	0.095568	0.10571	0.10713
Hexadecanethiol	0.092943	0.261	0.26443
Sugiol	0.088963	0.091036	0.090896
Oxononanal	0.084152	0.083908	0.083682
Allyl alcohol	0.081353	0.07923	0.078946
Stigmasteryl glucoside	0.079997	0.080042	0.080097
Dehydroabietic acid	0.079516	0.082589	0.082915
Cellobiose	0.077766	0.075732	0.075469
Allose	0.076979	0.074972	0.074702
Stigmasta diene 6 dione	0.072605	0.15929	0.16305
D Fructose	0.072299	0.070618	0.07042
Desmosterol	0.070943	0.069099	0.068879

Filbertone	0.070243	0.068408	0.068168
Tridecanoic acid	0.069893	0.070946	0.07083
Dodecane	0.06307	0.063175	0.06294
Testolactone	0.061583	0.064197	0.064539
Methylhexacosane	0.061233	0.059637	0.059424
Cycloartenol	0.061233	0.062623	0.06381
Tetradecanoic acid	0.057953	0.062201	0.062907
Uric acid	0.051611	0.050373	0.050344
Tetrahydrolycopene	0.048549	0.047285	0.047176
Vitamin D3	0.047325	0.046249	0.046069
Methyl d glucose	0.046275	0.045062	0.044912
all trans Retinoic acid	0.042426	0.045132	0.044955
Rhamnitol	0.042338	0.041228	0.041098
Cholestanol	0.04177	0.040692	0.041037
Phenylpyridine	0.040851	0.039801	0.039647
Cymbopogonol	0.037965	0.039156	0.039169
Cholic acid	0.036827	0.037802	0.03778
Palmitoylsn glycerol	0.03499	0.035125	0.036118
Dimethylpyrazole	0.034116	0.033298	0.033172
Paromomycin	0.033853	0.033021	0.032891
Melezitose	0.033678	0.032795	0.032667
Glycocholic acid	0.033503	0.032765	0.032742
Capric acid	0.033241	0.033195	0.0331
Methyl tetradecanoate	0.031623	0.033773	0.035342
Pregnenolone	0.031579	0.030756	0.030686
gamma Tocopherol	0.029654	0.030683	0.030737
Petasalbin	0.029567	0.028794	0.02872
Lysylvaline	0.029479	0.028927	0.029277
Ethanethioic acid	0.027905	0.02745	0.027668
cis Sesquisabinene hydrate	0.027467	0.027233	0.027233
Aspidospermidine	0.025368	0.042498	0.045729

Palmitoleic acid	0.024668	0.024338	0.024448
Methyltetradecane	0.024493	0.025544	0.025663
Undecenal	0.023531	0.023143	0.023335
isosorbide	0.022613	0.022921	0.023413
Melibiose	0.020732	0.020354	0.020581
Ribavirin	0.020557	0.021108	0.021096
Panaxydol	0.017539	0.017165	0.017098
Erucic acid	0.01732	0.016926	0.016899
Trigonelline	0.017277	0.016836	0.0168
Glycine	0.016577	0.018394	0.01922
Galactose	0.015308	0.014907	0.014861
lupinine	0.015133	0.015607	0.015582
undecenedioic acid	0.014609	0.014352	0.014495
Sinigrin	0.013996	0.014133	0.01481
Longispinogenin	0.013952	0.01444	0.014442
Eicosapentaenoic acid	0.013253	0.013295	0.013455
Ascaridole	0.012684	0.016055	0.017617
Ursodeoxycholic acid	0.011678	0.011376	0.01136
Brassicasterol	0.011328	0.011033	0.011031
Pentadecanoic acid	0.010935	0.074299	0.087097
Cineole	0.010935	0.010709	0.010915
Nonanal	0.010278	0.010046	0.035207
Xanthotoxol arabinoside	0.0098411	0.012066	0.016337
Campesterol	0.0096661	0.009438	0.009801
Pregnanediol	0.009185	0.010443	0.010542
undecenoic acid	0.00901	0.010052	0.011538
Pelargonic acid	0.0069981	0.008077	0.008048
Deoxy D glucitol	0.0069106	0.006858	0.006858
Geranylgeranyl PP	0.0067357	0.008028	0.009761
hydroxylycopene	0.0065607	0.017629	0.017606
D Glucose	0.0054673	0.006028	0.006414

Phosphonoacetate	0.0043301	0.004242	0.004228
solasodine beta D glucopyranoside	0.0040239	0.003948	0.003935
Acetoxy hydroxy heptadecenone	0.003674	0.021761	0.035234
Ethoxyquin	0.0032366	0.003173	0.003163
Undecanoic acid	0.0027774	0.009874	0.010009
Myristoleic acid	0.00087476	0.001563	0.002097
Hydroxyandrostene	0.00065607	0.021849	0.023737
Monoethylhexyl phthalic acid	3.55E-18	0.012593	0.012918
Isophthalamide	3.55E-18	0.00489	0.007675
Ethyl octadienoate	1.78E-18	0.003793	0.00378
Calcitriol	8.89E-19	0.005044	0.008584
tertbutyl benzenediol	8.89E-19	0.004599	0.00518
Stearaldehyde	8.89E-19	0.004599	0.00518
Phenylethyl beta D glucopyranoside	8.89E-19	0.003769	0.0039
Isopentacosane	8.89E-19	0.003389	0.003471
Methyl undecenoate	8.89E-19	0.003351	0.003397
Furoic acid	8.89E-19	0.00324	0.003283
Ethylbenzoic acid	8.89E-19	0.001955	0.002055
Methylhistamine	8.89E-19	0.00194	0.001979
Smilagenone	8.89E-19	0.001205	0.001494
Sakebiose	8.89E-19	0.001192	0.003133
Acetyloxy triphenylstannane	8.89E-19	0.001013	0.004133
Chlorozotocin	4.44E-19	0.003756	0.004252
Pteleine	4.44E-19	0.00188	0.002037
Dodecenoic acid	1.11E-19	0.00015	0.000306
Deoxyspergualin	0	0.006634	0.007679
Palmitoyl chloride	0	0.006506	0.018475
Cresol	0	0.003232	0.003344
